# HDAC6 modulates myofibril stiffness and diastolic function of the heart

**DOI:** 10.1172/JCI148333

**Published:** 2022-05-16

**Authors:** Ying-Hsi Lin, Jennifer L. Major, Tim Liebner, Zaynab Hourani, Joshua G. Travers, Sara A. Wennersten, Korey R. Haefner, Maria A. Cavasin, Cortney E. Wilson, Mark Y. Jeong, Yu Han, Michael Gotthardt, Scott K. Ferguson, Amrut V. Ambardekar, Maggie P.Y. Lam, Chunaram Choudhary, Henk L. Granzier, Kathleen C. Woulfe, Timothy A. McKinsey

**Affiliations:** 1 Department of Medicine, Division of Cardiology, and; 2 Consortium for Fibrosis Research & Translation, University of Colorado Anschutz Medical Campus, Aurora, Colorado, USA.; 3 Department of Proteomics, Novo Nordisk Foundation Center for Protein Research, Faculty of Health and Medical Sciences, University of Copenhagen, Copenhagen, Denmark.; 4 Department of Cellular and Molecular Medicine and Sarver Molecular Cardiovascular Research Program, University of Arizona, Tucson, Arizona, USA.; 5 Neuromuscular and Cardiovascular Cell Biology, Max Delbrück Center for Molecular Medicine in the Helmholtz Association, Berlin, Germany.; 6 Charité–Universitätsmedizin Berlin, Berlin, Germany.; 7 Cardiovascular and Pulmonary Research Laboratory, Department of Medicine, University of Colorado Anschutz Medical Campus, Aurora, Colorado, USA.

**Keywords:** Cardiology, Heart failure

## Abstract

Passive stiffness of the heart is determined largely by extracellular matrix and titin, which functions as a molecular spring within sarcomeres. Titin stiffening is associated with the development of diastolic dysfunction (DD), while augmented titin compliance appears to impair systolic performance in dilated cardiomyopathy. We found that myofibril stiffness was elevated in mice lacking histone deacetylase 6 (HDAC6). Cultured adult murine ventricular myocytes treated with a selective HDAC6 inhibitor also exhibited increased myofibril stiffness. Conversely, HDAC6 overexpression in cardiomyocytes led to decreased myofibril stiffness, as did ex vivo treatment of mouse, rat, and human myofibrils with recombinant HDAC6. Modulation of myofibril stiffness by HDAC6 was dependent on 282 amino acids encompassing a portion of the PEVK element of titin. HDAC6 colocalized with Z-disks, and proteomics analysis suggested that HDAC6 functions as a sarcomeric protein deacetylase. Finally, increased myofibril stiffness in HDAC6-deficient mice was associated with exacerbated DD in response to hypertension or aging. These findings define a role for a deacetylase in the control of myofibril function and myocardial passive stiffness, suggest that reversible acetylation alters titin compliance, and reveal the potential of targeting HDAC6 to manipulate the elastic properties of the heart to treat cardiac diseases.

## Graphical abstract

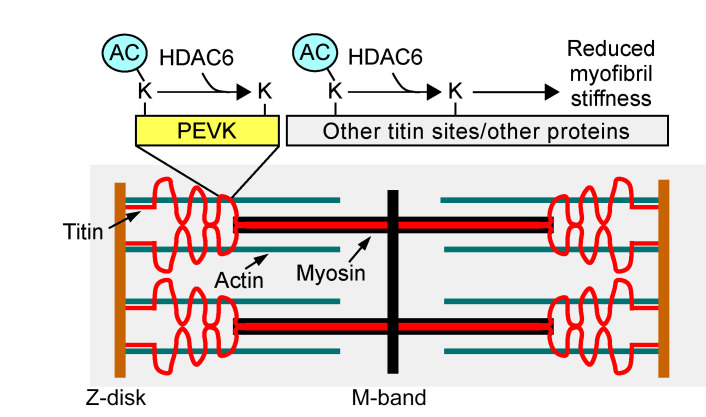

## Introduction

Stiffness of the heart is categorized as either active or passive. Active stiffness is dependent on actomyosin cross-bridge interactions, while passive stiffness is determined by myocardial extracellular matrix and titin (1). Modulation of titin compliance is linked to human cardiac disease (2), with decreased titin-based passive stiffness observed in patients with systolic dysfunction and dilated cardiomyopathy (DCM) (3–6), and titin stiffening associated with diastolic dysfunction (DD) and heart failure with preserved ejection fraction (HFpEF) (7–10). Increased compliance of titin in DCM impairs active contraction and sarcomere length–dependent tension generation (the Frank-Starling mechanism) (11, 12), while excessive titin stiffening in HFpEF diminishes ventricular filling during diastole (2, 8). Given the central role of titin in the control of cardiac homeostasis and disease, there is intense interest in developing therapeutic modalities with the capacity to negatively or positively tune titin compliance.

Titin, a modular protein that spans from the Z-disk to the M-band of the sarcomere, is integral to the regulation of myofibril passive stiffness. Titin is initially expressed as a fetal isoform of N2BA. In adults, RBM20-mediated splicing favors the expression of a shorter N2BA, which is gradually replaced by the even shorter N2B (Supplemental Figure 1; supplemental material available online with this article; https://doi.org/10.1172/JCI148333DS1) (13–16). Both isoforms consist of an amino-terminal Z-disk–binding region followed by an I-band spring region, the latter of which is the primarily subjected to differential splicing. Both titin isoforms harbor a common N2B element within the spring region. However, the spring region of the N2BA isoform contains more immunoglobulin-like (Ig-like) domain segments and a longer PEVK element, so named because it is rich in proline, glutamic acid, valine, and lysine residues. As such, with an extended spring region, the N2BA isoform is more compliant than the adult N2B isoform of titin. Other domains that are common to both isoforms of titin comprise the myosin-binding A-band, and the M-band, which binds myomesin (Supplemental Figure 1) (2). Altered titin N2BA/N2B expression ratio is associated with certain forms of heart failure (HF) in humans (2).

Another mechanism for regulation of titin compliance is through differential phosphorylation, with the spring elements of titin (N2B and PEVK) serving as hotspots for phosphorylation by kinases such as protein kinase A (PKA), protein kinase G (PKG), protein kinase C (PKC), extracellular signal–regulated kinase (ERK), and Ca^2+^/calmodulin-dependent protein kinase II (CaMKII) (Supplemental Figure 1) (8). From a therapeutic perspective, most effort has focused on restoring titin phosphorylation by stimulating PKG activity via phosphodiesterase 5 (PDE5) inhibition or soluble guanylyl cyclase (sGC) activation as an approach to decrease passive stiffness of the heart to improve relaxation in patients with HFpEF (17, 18).

Other posttranslational modifications of titin, including oxidation, glutathionylation, and sulfenylation, have been linked to the regulation of myofibril stiffness. Additionally, deacetylation of titin by NAD-dependent sirtuins was recently found to be associated with increased titin compliance (19). However, the degree to which reversible acetylation on lysine residues regulates titin-mediated passive stiffness remains unclear.

Here, we demonstrate that the zinc-dependent deacetylase histone deacetylase 6 (HDAC6) tightly regulates cardiac myofibril compliance, and we provide evidence to suggest that this effect is mediated by alterations in titin acetylation. HDAC6 inhibition leads to myofibril stiffening, while enhancement of HDAC6 activ ity increases myofibril compliance, both through mechanisms that are dependent on the PEVK element of titin. HDAC6 is currently being aggressively pursued as a therapeutic target for multiple indications, including cancer and neurodegenerative diseases, by virtue of its ability to regulate processes such as protein turnover and mitochondrial transport (20). Our data suggest that future studies of HDAC6 inhibitors in the clinic should incorporate evaluation of cardiac function, particularly diastolic performance. Beyond this, the findings presented here highlight a path toward the development of innovative treatments for HF, with HDAC6 inhibition and subsequent myofibril stiffening potentially ameliorating cardiac diseases marked by excessive titin compliance and systolic dysfunction, and HDAC6 gain of function benefiting patients with DD that is associated with titin stiffening.

## Results

### HDAC6 loss of function leads to titin stiffening.

**Figure 1:**
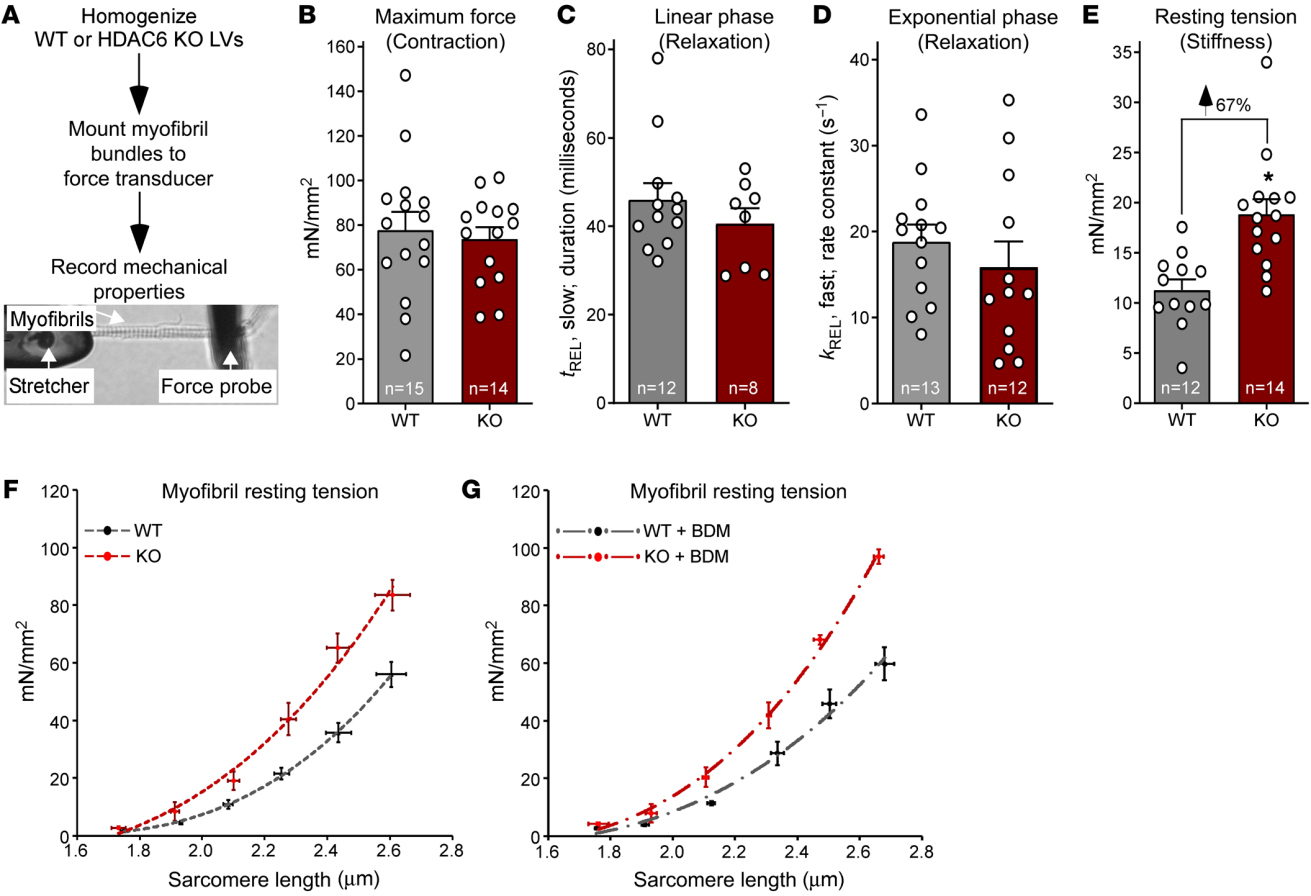
HDAC6 KO increases cardiac myofibril stiffness. (**A**) Schematic representation of ex vivo myofibril mechanics system. Myofibrils from left ventricles (LVs) of 6-month-old male mice were evaluated. (**B**) Myofibril tension (mN/mm^2^) generation in response to maximal calcium (pCa 4.5). (**C** and **D**) Linear (*t*_REL_, slow) and exponential (*k*_REL_, fast) myofibril relaxation upon removal of calcium (pCa 9.0). (**E**) Myofibril resting tension (mN/mm^2^) at a sarcomere length of 2.0–2.2 μm. For **B**–**E**, dots represent data from individual myofibrils. Mean + SEM is shown; *P < 0.05 vs. WT based on unpaired, 2-tailed *t* test. (**F**) Myofibril resting tension–to–sarcomere length curves. (**G**) Myofibrils were treated with the myosin ATPase inhibitor butanedione monoxime (BDM; 50 mM) before assessment of resting tension at the given sarcomere lengths. For **F** and **G**, data are presented as mean ± SEM, fitted by third-order polynomials, from 4 animals per group, with 6–8 myofibrils per mouse analyzed.

Reversible acetylation of sarcomeric proteins is emerging as a mechanism for posttranslational regulation of cardiac function (21), but the roles of specific HDAC isoforms in the control of myofibrillar proteins remain unclear. Previously we demonstrated that mice lacking HDAC6, which serves prominent roles in the cytoplasm by deacetylating cytoskeletal proteins, have apparently normal cardiac function at baseline, but are protected from systolic dysfunction elicited by chronic angiotensin II administration or transverse aortic constriction (22). To further address the possibility that HDAC6 functions in the cytoplasm of cardiomyocytes to regulate mechanical properties of sarcomeres, bundles of sarcomeres (myofibrils), obtained from homogenized left ventricles (LVs) of WT and HDAC6-KO mice, were mounted on a microtool attached to a piezo motor and a cantilevered force probe, and their mechanical properties were quantified ex vivo (Figure 1A). Myofibrils from HDAC6-deficient mice generated force in response to Ca^2+^ and relaxed upon Ca^2+^ removal equivalently to those from hearts of WT mice (Figure 1, B–D, and Supplemental Figure 2). In contrast, myofibril resting tension, which is a measure of titin compliance and residual cross-bridge binding, was elevated in HDAC6-KO mice compared with WT controls (Figure 1E).

To expand the evaluation of the impact of HDAC6 on cardiac stiffness, myofibrils from WT and HDAC6-KO hearts were subjected to stepwise extension ex vivo, and concomitant alterations in resting tension were quantified to yield resting tension-to-sarcomere length curves (23). HDAC6 deletion resulted in a resting tension-to-sarcomere length curve in comparison with WT controls (Figure 1F). A similar effect was observed using myofibrils treated with the myosin ATPase inhibitor butanedione monoxime (BDM) (Figure 1G), ruling out the possible contribution of residual binding of cross-bridges to this phenotype, and further suggesting that HDAC6 deletion diminishes titin compliance.

**Figure 2:**
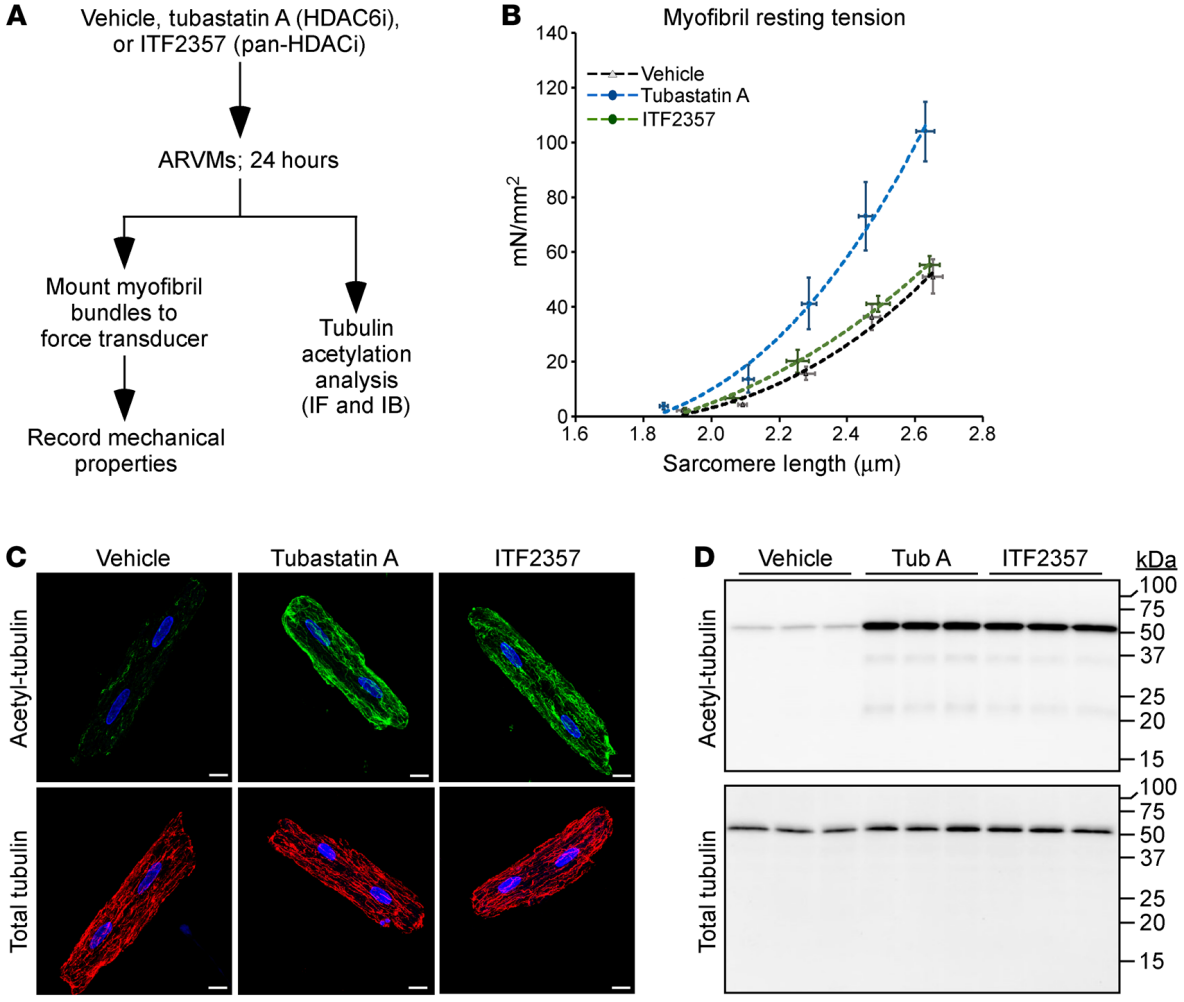
Selective pharmacological inhibition of HDAC6 increases cardiac myofibril stiffness. (**A**) Schematic representation of the adult rat ventricular myocyte (ARVM) experiment. (**B**) Resting tension–to–sarcomere length curves obtained with myofibrils isolated from ARVMs treated as indicated. Data are presented as mean ± SEM, fitted by third-order polynomials, from 4 separate ARVM preparations per group, with 6–8 myofibrils per preparation analyzed. (**C**) Indirect immunofluorescence analysis of acetyl-tubulin and total tubulin in ARVMs; scale bars: 10 μm. (**D**) Immunoblot analysis of acetyl-tubulin and total tubulin in whole-cell homogenates from treated ARVMs. See complete unedited blots in the supplemental material.

To determine whether HDAC6 catalytic activity regulates myofibril stiffness, cultured adult rat ventricular myocytes (ARVMs) were treated for 24 hours with the HDAC6-selective inhibitor tubastatin A (20); ITF2357, which inhibits HDAC6 as well as several other zinc-dependent HDACs; or vehicle control (Figure 2A). Consistent with the findings from KO hearts, myofibrils from ARVMs exposed to tubastatin A exhibited elevated resting tension (Figure 2B). Surprisingly, ITF2357 had no effect of myofibril stiffness. HDAC6 is a tubulin deacetylase (24), and pharmacodynamic assessment of tubulin acetylation as a marker of HDAC6 inhibition confirmed that tubastatin A and ITF2357 inhibited HDAC6 equivalently in ARVMs (Figure 2, C and D). Thus, enhanced myofibril stiffness is only observed upon selective inhibition of HDAC6. These findings agree with the prior demonstration that ITF2357 ameliorates rather than exacerbates DD in murine models (21, 25).

### HDAC6 gain of function leads to decreased titin stiffness.

**Figure 3:**
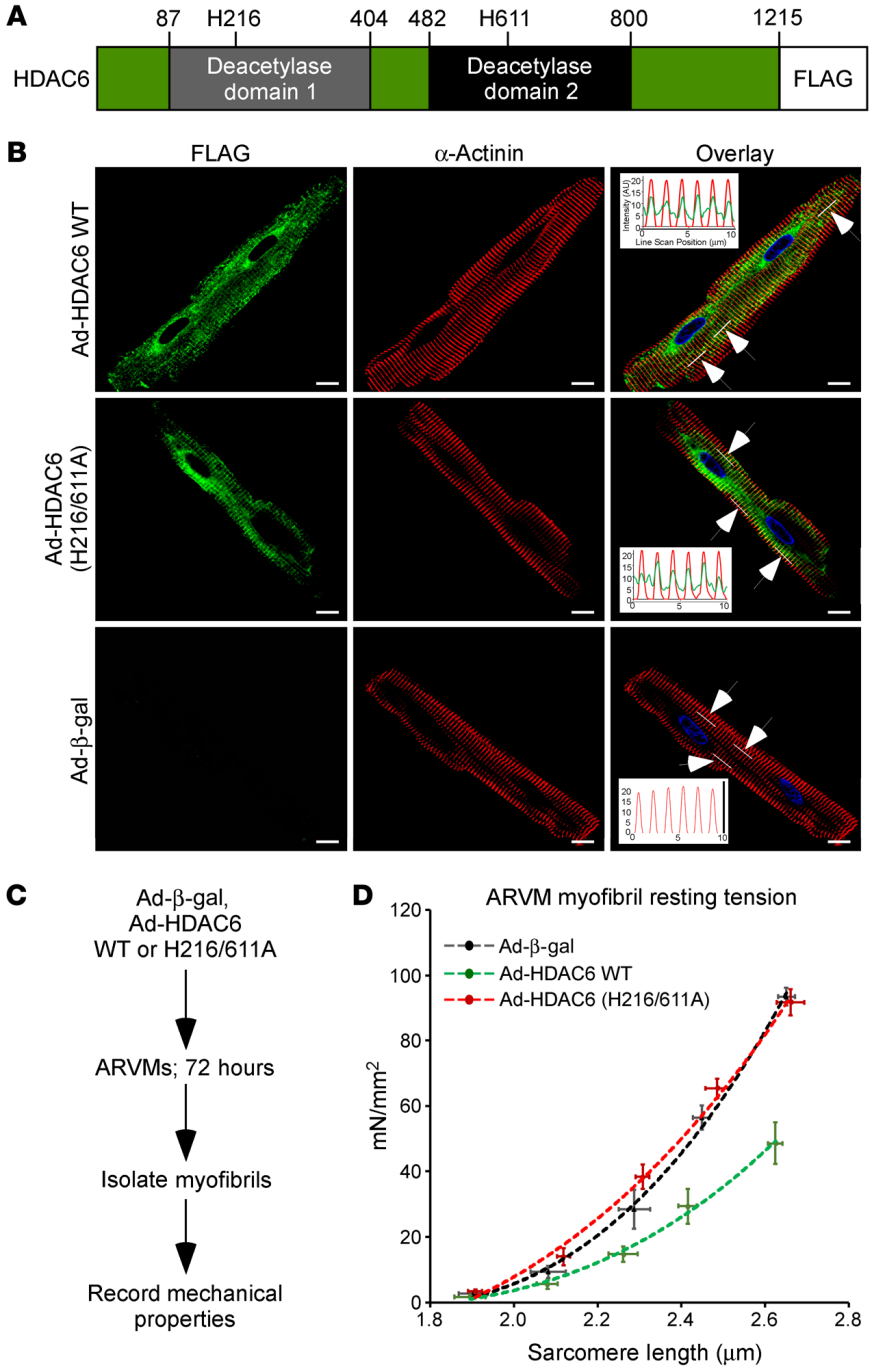
Ectopic HDAC6 colocalizes with cardiomyocyte sarcomeres and reduces myofibril stiffness. (**A**) Schematic representation of HDAC6 with amino acid numbers indicated. (**B**) Indirect immunofluorescence of ARVMs infected with adenoviruses encoding FLAG-tagged WT HDAC6, HDAC6 harboring 2 amino acid substitutions that abolish enzymatic activity (H216/611A), and β-galactosidase (β-gal) as a negative control. Insets in the overlay are line scans of fluorescence intensity in the 488 nm and 568 nm channels within the regions of the cells indicated by the white lines (arrows point to white lines). Averages from the 3 regions are shown, and overlapping peaks of fluorescence reveal colocalization of HDAC6 and sarcomeric α-actinin. DAPI fluorescence of nuclei is also shown. Scale bars: 10 μm. (**C**) Schematic representation of the ARVM myofibril experiment using adenoviruses. (**D**) Myofibril resting tension–to–sarcomere length curves. Data are presented as mean ± SEM, fitted by third-order polynomials, from 4 animals per group, with 6–8 myofibrils per mouse analyzed.

Unlike other HDACs, HDAC6 contains tandem deacetylase domains (Figure 3A). To further address the function of HDAC6 in the heart, ARVMs were infected with adenoviruses expressing wild-type HDAC6 (Ad-HDAC6 WT), derivatives of the enzyme harboring amino acid substitutions that abolish catalytic activity (Ad-HDAC6 H216A, H611A, or H216/611A), or a β-galactosidase negative control (Ad-β-gal). Immunoblotting confirmed efficient expression of ectopic HDAC6 in the cultured adult cardiomyocytes (Supplemental Figure 3A). In agreement with prior findings (26, 27), deacetylase domain 2 of HDAC6 was required for deacetylation of endogenous α-tubulin (Supplemental Figure 3, A and B), establishing the validity of this cell-based, ectopic expression system. Consistent with a role for HDAC6 in controlling myofibril function, confocal imaging revealed a pool of the enzyme that colocalized with sarcomeric α-actinin at the Z-disk in cardiomyocytes, independently of its catalytic activity (Figure 3B and Supplemental Figure 3C). To determine whether HDAC6 gain of function alters passive stiffness of cardiomyocytes, ARVMs were infected with the adenoviruses for 72 hours, and myofibrils were subsequently isolated for evaluation of mechanics (Figure 3C). Strikingly, ectopic expression of HDAC6 dramatically increased the compliance of cardiac myofibrils in a manner dependent on the catalytic activity of deacetylase domain 2 (Figure 3D and Supplemental Figure 3D).

**Figure 4:**
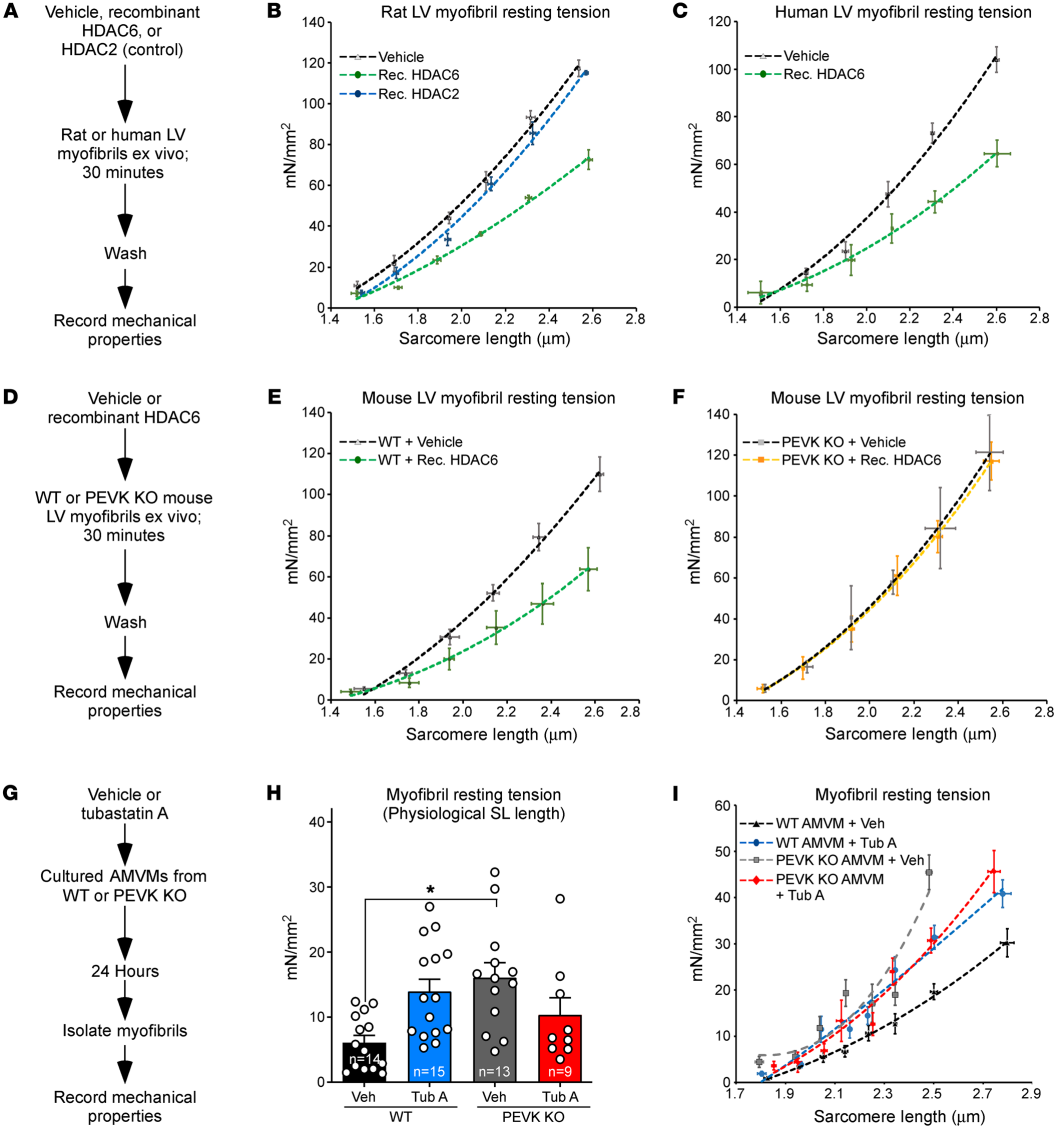
Recombinant HDAC6 regulates myofibril stiffness in a manner that is dependent on a region of titin encompassing a portion of the PEVK element and an adjacent Ig-like domain. (**A**) Schematic representation of the ex vivo assay using recombinant HDACs and myofibrils isolated from rat or human left ventricles (LVs). (**B** and **C**) Myofibril resting tension–to–sarcomere length curves. (**D**) Schematic representation of the ex vivo assay using myofibrils from WT and PEVK/Ig-like domain–KO mouse LVs. (**E** and **F**) Myofibril resting tension–to–sarcomere length curves. (**G**) Schematic representation of the experiment using cultured adult mouse ventricular myocytes (AMVMs) from WT and PEVK-KO mice. (**H**) Myofibril resting tension (mN/mm^2^) at a sarcomere length of 2.0–2.2 μm. (**I**) Myofibril resting tension–to–sarcomere length curves. For **B**, **C**, **E**, **F**, and **I**, data are presented as mean ± SEM, fitted by third-order polynomials. For **E**, **F**, **H**, and **I**, 6–8 myofibrils from 3 mice per group (WT and KO) were analyzed. For **H**, mean + SEM is shown; *P < 0.05 vs. WT + vehicle based on 1-way ANOVA with Tukey’s multiple-comparison test.

Ex vivo incubation of purified rat myofibrils with recombinant HDAC6, but not HDAC2, reduced titin stiffness (Figure 4, A and B), establishing the ability of HDAC6 to directly and selectively modulate sarcomere function; HDAC2 was chosen as a control for specificity because of our prior demonstration that the enzyme associates with cardiac myofibrils to regulate relaxation (21). Recombinant HDAC6 also increased compliance of human myofibrils obtained from nonfailing donor LV explants, illustrating a conserved ability of this deacetylase to control cardiomyocyte passive stiffness in higher mammals (Figure 4C).

### The PEVK region of titin is required for HDAC6-mediated modulation of myofibril stiffness.

To begin to determine whether HDAC6-mediated alterations in myofibril stiffness are governed via titin, recombinant HDAC6 was incubated with myofibrils obtained from adult mouse hearts from WT mice or mice in which a portion (amino acids 12717–12998) of the PEVK element and an adjacent Ig-like domain of titin were deleted by homologous recombination (Figure 4D) (28); these PEVK residues are expressed in all titin isoforms. As with samples obtained from rat and human hearts, recombinant HDAC6 efficiently reduced the stiffness of mouse cardiac myofibrils (Figure 4E). Remarkably, myofibrils from mice lacking 282 amino acids of the PEVK/Ig-like region of titin were completely resistant to HDAC6 (Figure 4F). Notably, the acetylation state of cardiac α-tubulin was unaffected by deletion of the PEVK domain, arguing against an indirect role for this region of titin in the control of HDAC6 activity (Supplemental Figure 4, A and B).

Next, we sought to determine whether myofibril stiffening upon HDAC6 inhibition is dependent on the PEVK domain of titin. Adult mouse ventricular myocytes (AMVMs) were obtained from WT and PEVK-KO animals and cultured in the absence or presence of tubastatin A before isolation of myofibrils for mechanical measurements (Figure 4G). In line with data obtained with ARVMs, pharmacological inhibition of HDAC6 with tubastatin A led to an increase in myofibril resting tension in WT AMVMs (Figure 4H). However, the HDAC6 inhibitor failed to augment passive stiffness of PEVK-KO myofibrils; PEVK-KO myofibrils had higher basal resting tension than WT controls (Figure 4I), which is consistent with the previously defined role for this region of titin in the regulation of myofibril compliance (28). Analysis of resting tension-to-sarcomere length curves confirmed that tubastatin A–mediated stiffening of myofibrils was dependent on the PEVK region of titin (Figure 4I). Together, these findings define a critical role for the PEVK spring element of titin in HDAC6-mediated regulation of cardiomyocyte passive stiffness.

### HDAC6 reverses PKC-mediated titin stiffening in human myofibrils.

**Figure 5:**
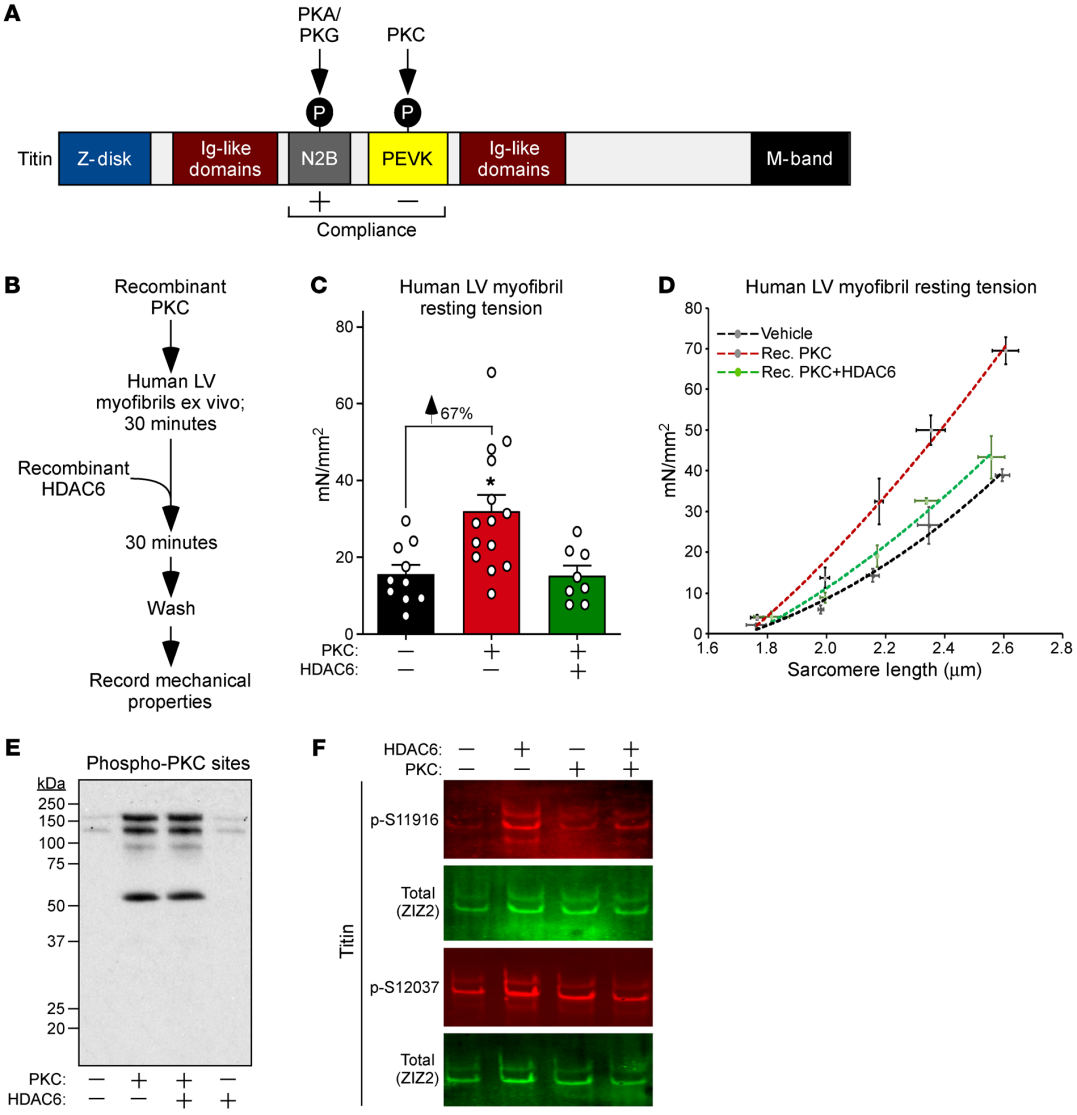
HDAC6 reverses PKC-mediated stiffening of human myofibrils. (**A**) Schematic representation of titin, with the impact of phosphorylation of the N2B and PEVK regions indicated. (**B**) Schematic representation of the ex vivo assay using human myofibrils and recombinant forms of PKCα and HDAC6. (**C**) Myofibril resting tension measurements at physiological sarcomere length (~2.19 μm). Mean + SEM is shown; *P < 0.05 vs. untreated myofibrils based on 1-way ANOVA with Tukey’s multiple-comparison test. (**D**) Myofibril resting tension–to–sarcomere length curves. Data are presented as mean ± SEM, fitted by third-order polynomials. Myofibrils from 3 nonfailing human hearts were each treated with vehicle, recombinant PKCα, or recombinant PKCα followed by recombinant HDAC6; 6–8 myofibrils per treatment were analyzed and averaged per heart. (**E**) Immunoblotting was performed with an anti–PKC substrates antibody and solubilized proteins from myofibrils treated as indicated. (**F**) Titin was immunoblotted with antibodies specific for phospho-S11916 (S11878 in human) and phospho-S12037 (S12022 in human) in the PEVK domain, as well as an antibody against the titin Z1Z2 element to assess total titin levels.

PKC-dependent phosphorylation of the PEVK element of titin leads to myofibril stiffening (29), while PKA- and PKG-mediated phosphorylation of the adjacent N2B region increases titin compliance (Figure 5A) (30, 31). Immunoblotting of mouse LV homogenates using phospho-specific antibodies failed to reveal an impact of HDAC6 deletion on PEVK or N2B phosphorylation (Supplemental Figure 5, A and B), suggesting that HDAC6 regulates titin stiffness by directly deacetylating the protein as opposed to indirectly affecting its phosphorylation state.

Elevated PEVK phosphorylation is associated with increased cardiomyocyte passive stiffness in preclinical models and in human HF (8). To begin to address the therapeutic potential of HDAC6 gain of function, an ex vivo experiment was performed to determine whether HDAC6 is capable of overriding the cardiac stiffening effect of PKC. Myofibrils obtained from nonfailing human LVs were pretreated with recombinant PKCα, with or without the addition of recombinant HDAC6 (Figure 5B). PKCα dramatically increased myofibril resting tension at physiological sarcomere length (2.0–2.2 μm), and, remarkably, this stiffening was completely normalized upon subsequent exposure of the myofibrils to HDAC6 (Figure 5C). Recombinant HDAC6 also largely blocked the PKC-induced steeper resting tension-to-sarcomere length curve (Figure 5D). The presence of recombinant HDAC6 did not reduce PKC-driven phosphorylation of cardiac proteins and, if anything, may have augmented titin phosphorylation (Figure 5, E and F). These data demonstrate the ability of HDAC6 to neutralize PKC-mediated stiffening of human myofibrils through a mechanism that is independent of phosphorylation, suggesting that HDAC6 gain of function could be therapeutically beneficial in the setting of DD.

### HDAC6 deletion alters cardiac myofibrillar protein acetylation.

To further address the mechanism(s) by which HDAC6 modulates myofibril stiffness, the acetylation state of sarcomeric proteins was evaluated. Immunoblotting demonstrated that reduced myofibril compliance in HDAC6-KO mice was not associated with changes in titin isoform expression or the global acetylation state of titin (Supplemental Figure 6, A–F). Next, mass spectrometry–based acetylproteomics analysis was performed on LV lysates to address the impact of HDAC6 deletion on site-specific lysine acetylation in the heart. Online liquid chromatography–coupled tandem mass spectrometry was performed with affinity-purified acetylated peptides derived from trypsin-digested total LV protein homogenates and revealed an increase in acetylation of several lysines in titin in HDAC6-KO hearts, including 2 sites near the PEVK element (K13013 and K13597) (Supplemental Figure 7A). Acetylation of K191 of cardiac myosin-binding protein C (MyBP-C) was also increased in HDAC6-KO hearts (Supplemental Figure 7A). Annotated MS2 spectra confirmed the identity of specific lysine acetylation sites in titin and MyBP-C (Supplemental Figure 7, B–D). These findings suggest that HDAC6 deacetylates multiple lysine residues within cardiac sarcomeric proteins, and support a role for HDAC6 as a titin deacetylase.

### HDAC6 loss of function exacerbates DD in mouse models.

**Figure 6:**
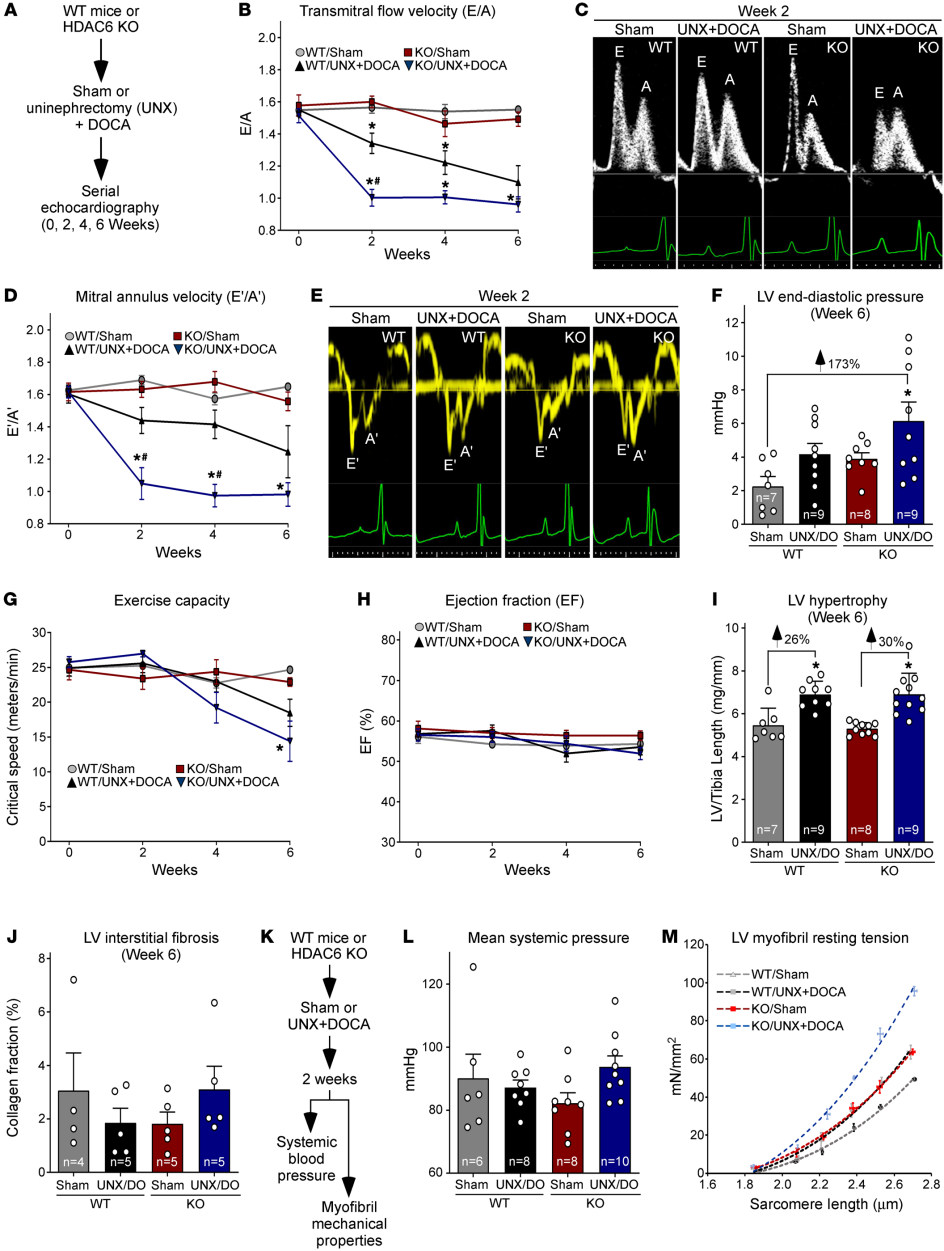
HDAC6 deletion exacerbates DD and cardiac myofibril stiffening in the UNX/DOCA model. (**A**) Schematic representation of mouse model of DD with preserved ejection fraction. (**B**) Serial Doppler echocardiographic measurements of mitral inflow velocity (E/A), a parameter of diastolic cardiac function. (**C**) Representative E/A images. (**D**) Serial echocardiographic measurements of septal mitral annulus velocity (E′/A′), another measure of diastolic function. (**E**) Representative E′/A′ images. (**F**) Invasive, catheter-based measurements of LV end-diastolic pressure at study endpoint (6 weeks). Mean + SEM values are shown and were compared by 2-way ANOVA with Tukey’s multiple-comparison test; *P < 0.05 vs. WT/sham. (**G**) Critical speed was determined as a measure of exercise capacity. (**H**) Echocardiographic assessment of systolic function as determined by ejection fraction. For **B**, **D**, **G**, and **H**, mean ± SEM values are shown and were compared by a mixed-effects model for repeated measures with Tukey’s multiple-comparison test; *P < 0.05 vs. WT/sham, ^#^*P* < 0.05 vs. WT/UNX + DOCA; animal numbers for each time point are provided in Supplemental Table 1. Echocardiographic data are summarized in Supplemental Table 2. (**I**) LV–to–tibia length assessment of cardiac hypertrophy upon necropsy. Mean + SEM values are shown and were compared by 1-way ANOVA with Tukey’s multiple-comparison test; *P < 0.05 vs. corresponding sham control. (**J**) LV sections were stained with Picrosirius red dye, and the ratio of positively stained (red) pixels to the total pixel number of each section (collagen fraction %) was calculated. Mean values are shown (+ SEM). Two-way ANOVA revealed no significant difference between groups. (**K**) Schematic representation of the 2-week study to assess the impact of HDAC6 deletion on blood pressure and myofibril stiffness in the mouse model of DD with preserved ejection fraction. (**L**) Tail cuff measurements of mean systemic pressure (mmHg). Data are presented as mean + SEM. (**M**) Myofibril resting tension–to–sarcomere length curves. Data are presented as mean ± SEM, fitted by third-order polynomials, from 4 animals per group, with 6–8 myofibrils per mouse analyzed.

We hypothesized that DD is exacerbated by HDAC6 deletion as a result of elevated passive stiffness of the heart. To test this, HDAC6-KO mice and WT controls were evaluated in a model of hypertension-induced DD with preserved ejection fraction driven by combined uninephrectomy (UNX) and deoxycorticosterone acetate (DOCA) (Figure 6A and Supplemental Table 1). Serial Doppler echocardiography was used to quantify diastolic function by measuring the ratio of the early filling (E) phase of the LV during diastole, which is due to relaxation of the LV, and the late filling (A) phase, which is mediated by contraction of the atrium. Two and four weeks after UNX/DOCA, HDAC6-KO exhibited more severe DD than WT controls, as evidenced by pronounced reduction in E/A (Figure 6, B and C). Doppler measurements of septal mitral annulus velocity (E′/A′) confirmed the more rapid onset of DD in HDAC6-KO mice compared with WT controls (Figure 6, D and E). At the study endpoint of 6 weeks, E/A and E′/A′ were equivalent between HDAC6-KO and WT mice. Nonetheless, invasive hemodynamic measurements obtained at this time confirmed that HDAC6-KO mice subjected to UNX/DOCA had elevated LV end-diastolic pressure compared with controls (Figure 6F), as well as a greater degree of exercise intolerance (Figure 6G), which correlates with DD (32). WT and HDAC6-KO mice had preserved ejection fraction throughout the 6-week study (Figure 6H).

DD is often attributed to cardiac hypertrophy and fibrosis. However, HDAC6-KO and WT mice developed equivalent hypertrophy in response to UNX/DOCA, as determined by LV mass–to–tibia length measurements and echocardiographic assessment of LV wall thickness (Figure 6I and Supplemental Table 2). Picrosirius red staining of LV sections failed to reveal significant interstitial fibrosis in any of the groups (Figure 6J).

To further address the mechanism of more severe DD in HDAC6-KO mice, a repeat study was performed, with analyses focusing on the 2-week time point, when the difference in DD between WT and HDAC6-KO mice was most exaggerated (Figure 6K). Tail cuff measurements revealed that mice subjected to UNX/DOCA remained normotensive 2 weeks after surgery (Figure 6L), demonstrating that the observed DD at this early stage occurred independently of high blood pressure. In contrast, resting tension-to-sarcomere length curves showed that UNX/DOCA treatment for 2 weeks led to stiffening of LV myofibrils, and the reduction in myofibril compliance was exaggerated in mice lacking HDAC6 (Figure 6M). These data suggest that the intensified DD in HDAC6-KO mice is due to increased stiffening of titin.

Given that aging is an independent risk factor for the development of DD, we also performed serial echocardiography on normotensive WT and HDAC6-KO mice over the course of 18 months. Ejection fraction was preserved in WT and HDAC6-KO mice throughout the duration of the study (Supplemental Figure 8A). However, consistent with findings from the UNX/DOCA model, HDAC6-KO mice exhibited signs of impaired diastolic function with aging compared with WT controls (Supplemental Figure 8, B and C, and Supplemental Table 3). Evaluation of a greater number of mice, and with more advanced age, will be required to corroborate these findings.

## Discussion

**Figure 7:**
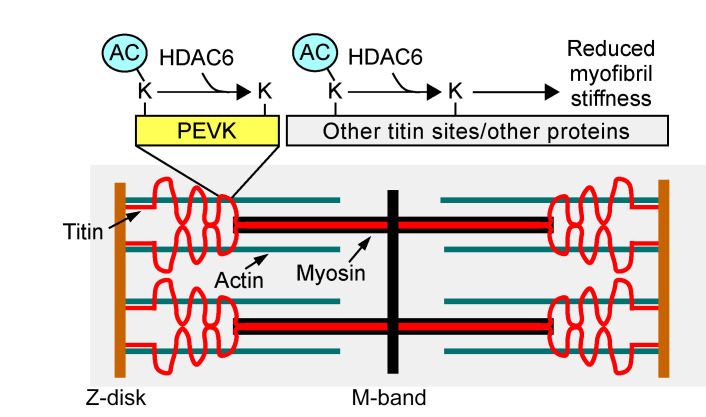
A model for the regulation of titin stiffness by HDAC6. HDAC6-mediated deacetylation reduces myofibril stiffness, whereas inhibition of HDAC6-mediated deacetylation leads to myofibril stiffening. We propose that HDAC6 regulates myofibril compliance, at least in part, through deacetylation of lysines embedded within, or adjacent to, the PEVK element of titin, and possibly through deacetylation of other regions of titin and/or other sarcomeric proteins.

By identifying HDAC6 as a regulator of myofibril stiffness, the current study provides evidence to support a role for reversible lysine acetylation in the control of the elastic properties of titin, and titin-based passive stiffness of the myocardium. We initially established a putative connection between HDAC6 and titin by evaluating the mechanical properties of isolated myofibrils from WT and HDAC6-KO mice ex vivo. Deletion of HDAC6 augmented myofibril stiffness in a manner that persisted in the presence of BDM, which disrupts actomyosin cross-bridges, strongly suggesting that HDAC6 modulates myofibril compliance via titin. Conversely, treatment of murine or human myofibrils with recombinant HDAC6 led to enhanced compliance. Importantly, the ability of HDAC6 to alter myofibril elastic properties was lost upon deletion of 282 amino acids of titin encompassing the approximately 190 carboxy-terminal residues of the PEVK element and an adjacent Ig-like domain. Thus, we propose that HDAC6 regulates myofibril compliance, at least in part, through deacetylation of lysines embedded within, or adjacent to, this region of titin, and possibly through deacetylation of other sarcomeric proteins (Figure 7).

Prior acetylproteomics studies have shown a number of titin lysine residues to be acetylated in rodent hearts (33, 34). Importantly, one of these sites corresponds to mouse K12905 near the PEVK/Ig-like region of titin that is necessary for recombinant HDAC6 to reduce myofibril stiffness. In addition, K12905 and 5 additional lysines in this region were found to be acetylated in skeletal muscle titin (34, 35), with one site (K12868 in mouse) showing elevated acetylation in response to Scriptaid (35), a hydroxamic acid HDAC inhibitor with a selectivity profile similar to that of ITF2357. It has been proposed that, upon phosphorylation, introduction of negatively charged phosphate groups to sites in the highly basic PEVK domain increases titin-based passive stiffness by altering ionic intramolecular interactions (8). It is possible that neutralization of the positive charge of lysine by acetylation in response to HDAC6 inhibition increases titin stiffness by further enhancing these intramolecular interactions. Consistent with this notion, using an ex vivo system with isolated myofibrils from human heart, we demonstrated that PKCα-mediated titin stiffening can be reversed by treatment of myofibrils with recombinant HDAC6.

Immunoblotting with anti–acetyl-lysine antibodies confirmed that cardiac titin is acetylated, but we failed to note a significant difference in this global titin acetylation signal between WT and HDAC6-KO hearts, suggesting that HDAC6 selectively deacetylates one or a small subset of the total acetylated lysines on titin. In support of this notion, subsequent mass spectrometry analysis revealed site-specific increases in titin acetylation in HDAC6-KO hearts compared with WT controls. Assessment of the impact of converting these lysines to arginine, which cannot be acetylated, or glutamine, which sometimes functions as an acetyl-lysine mimetic, will be required to assess their potential roles in the regulation of myofibril stiffness. It is also possible that HDAC6 regulates diastolic function by deacetylating sarcomeric proteins other than titin. In this regard, we observed an increase in MyBP-C acetylation in HDAC6-KO hearts compared with WT controls, and mutations in the gene encoding this protein have been shown to trigger DD (36). Implementation of more quantitative proteomics methods, such as stable isotope labeling by amino acids (SILAC), would yield a comprehensive inventory of lysines in titin and other myofibrillar proteins that are substrates for HDAC6-mediated deacetylation.

It is not known whether our findings are related to the recent demonstration that increasing NAD^+^ by administering its precursor, nicotinamide, ameliorates DD in association with reductions in titin stiffness and acetylation state in murine models (19). While nicotinamide is predicted to stimulate sirtuin activity, not HDAC6 activity, prior work has established that HDAC6 and sirtuins can function redundantly by deacetylating common substrates (37). Thus, it is possible that HDAC6 and sirtuins modulate myofibril stiffness through similar mechanisms.

A paradoxical finding from the current study is that tubastatin A, which selectively inhibits HDAC6, increases titin stiffness, while ITF2357, which inhibits HDAC6 as well as multiple other zinc-dependent HDACs, fails to alter the passive tension of myofibrils. This is not due to differences in potency or cellular activity of the compounds, since tubastatin A and ITF2357 inhibit HDAC6 with low-nanomolar potency, and both dramatically increased tubulin acetylation in ARVMs. We postulate that concomitant inhibition of numerous HDAC isoforms by ITF2357 neutralizes the myofibril stiffening action of HDAC6 inhibition, perhaps by enhancing acetylation of lysine residues that increase titin compliance. This scenario would be analogous to the mode in which kinases differentially modulate titin compliance based on the position of the target phospho-acceptor sites (8). Nonetheless, we cannot rule out the possibility that the inability of ITF2357 to stiffen myofibrils is governed by an alternative HDAC-dependent and/or off-target mechanism. We previously demonstrated that ITF2357 and the related pan-HDAC inhibitor suberoylanilide hydroxamic acid (SAHA) improve diastolic function in murine and feline models of hypertension, aging, and chronic pressure overload in association with enhanced myofibril relaxation (21, 38). The inability of pan-HDAC inhibition to alter titin compliance likely explains why ITF2357 and SAHA failed to exacerbate DD, and instead elicited protective actions in the heart through distinct mechanisms, such as enhancing the acetylation state of troponin I, which augments myofibril relaxation (39). The relative contributions of impaired myofibril relaxation and titin stiffening to the pathogenesis of DD remain to be determined.

It is important to note that changes in the viscoelastic properties of the microtubule network have recently been shown to contribute to cardiac stiffening and DD (40). Since HDAC6 is a tubulin deacetylase, it is conceivable that part of its ability to modulate diastolic function in vivo is mediated via microtubules. Nonetheless, it is improbable that the myofibril preparations used in our ex vivo assays contained intact microtubules, and thus the mechanical changes observed following HDAC6 inhibition or activation are likely due to direct effects on the acetylation state of titin or other sarcomeric proteins.

Therapeutic strategies designed to enhance titin compliance have focused on PKG activation and, more recently, on development of small-molecule RBM20 inhibitors and antisense oligonucleotides to promote expression of larger, more compliant titin isoforms (41, 42). Based on our findings, HDAC6 gain of function, either pharmacologically or through gene therapy–based ectopic expression of HDAC6 in cardiomyocytes, should be considered as an alternative or complementary approach to enhance titin compliance for the treatment of patients with DD. Conversely, DCM with systolic dysfunction is associated with increased myofilament compliance and submaximal force generation, leading to speculation that titin-stiffening agents could enhance contractility and have therapeutic benefit in the context of HF with reduced ejection fraction (HFrEF) (6). The possible utility of HDAC6 inhibition for the treatment of HFrEF is underscored by the prior demonstration that HDAC6 catalytic activity is elevated in models of HF (43), and HDAC6 KO and/or tubastatin A administration improves systolic function in mouse models of pressure overload, angiotensin II infusion, doxorubicin treatment, and ischemia/reperfusion (22, 44, 45). Furthermore, HDAC6 inhibition blocks the formation of cardiotoxic protein aggregates associated with desmin-related cardiomyopathy (46). Nonetheless, the extent to which titin alterations contribute to the efficacy observed in these models is unclear.

Evaluation of HDAC function in the heart has historically focused on epigenetic regulation of gene transcription through removal of acetyl groups from histone tails. However, to date, the most prominent functions of HDAC6 have been noted in the cytoplasm, where it controls the acetylation state of cytoskeletal proteins such as tubulin (24). Thus, HDAC6 is a particularly attractive target since its inhibition largely avoids untoward effects of broad-based epigenetic regulation throughout the body (47). Mouse knockout studies highlight this point. Despite having mild DD, as described here, mice with global deletion of HDAC6 develop normally and do not exhibit overt pathological phenotypes (48). In contrast, whole-body deletion of HDACs that serve fundamental roles in the control of histone deacetylation, such as HDAC1 and HDAC2, leads to early embryonic or perinatal mortality (49). Multiple HDAC6-selective inhibitors are in clinical development for indications such as cancer, neurodegeneration, and autoimmune diseases and, to our knowledge, have not elicited adverse effects in humans (20). Nonetheless, our findings suggest that HDAC6-selective inhibition should be avoided in individuals presenting with DD, since myofibril stiffening would be predicted to aggravate the ventricular filling defect. This concept is supported by the demonstration that HDAC6-KO mice developed more severe DD than controls in the UNX/DOCA model, and that HDAC6-KO mice have a propensity to develop exaggerated DD with aging compared with WT controls. Thus, at the level of cardiac myofilament, manipulation of HDAC6 can be both beneficial and detrimental, emphasizing the need to carefully titrate the function of this deacetylase, either positively or negatively, for therapeutic purposes.

### Limitations and conclusions.

A limitation of our study is that we did not demonstrate that HDAC6 directly deacetylates titin. Future identification of lysine acetylation sites that regulate myofibril stiffness will facilitate assessment of direct versus indirect effects of HDAC6 on titin compliance. Furthermore, although the mouse UNX/DOCA model is useful for studying DD in the context of preserved ejection fraction, the animals do not develop symptoms of overt HF. Thus, additional work is needed to determine whether HDAC6 inhibition exacerbates other hallmark features of HFpEF, such as lung edema.

In conclusion, our findings suggest a role for HDAC6-reversible acetylation as a posttranslational modification that controls myocardial passive stiffness. The data underscore emerging nongenomic roles for HDACs in the heart, and the potential to develop unique interventions for distinct forms of HF based on the ability to influence the elastic properties of titin by manipulating the catalytic activity of HDAC6.

## Methods

Further information can be found in Supplemental Methods.

### Myofibril mechanics analyses.

Myofibril mechanics were quantified using the fast solution switching technique (21, 50). Frozen LV sections were skinned in 0.5% Triton X-100 in rigor solution (132 mM NaCl, 5 mM KCl, 1 mM MgCl_2_, 10 mM Tris, 5 mM EGTA, pH 7.1) containing protease inhibitors (10 μM leupeptin, 5 μM pepstatin, 200 μM PMSF, and 10 μM E64) as well as 500 μM NaN_3_ and 500 μM DTT at 4°C overnight. Skinned LVs were washed in fresh rigor solution and homogenized (Tissue-Tearor, Thomas Scientific) in relaxing solution (pCa 9.0, where pCa = –log_10_[calcium concentration]) containing protease inhibitors. Myofibril suspensions were transferred to a temperature-controlled chamber (15°C) containing relaxing solution (pCa 9.0; 100 mM Na_2_EGTA, 1 M potassium propionate, 100 mM Na_2_SO_4_, 1 M MOPS, 1 M MgCl_2_, 6.7 mM ATP, and 1 mM creatine phosphate, pH 7.0). Myofibril bundles were mounted between 2 microtools. One tool was connected to a motor that could produce rapid length changes (Mad City Labs). The second tool was a calibrated cantilevered force probe (10.75 μm/μN; frequency response 2–5 kHz) . Myofibril length was set at approximately 2.2 μm. Average sarcomere lengths and myofibril diameters were measured using ImageJ software (NIH). Mounted myofibrils were activated and relaxed by rapid translation of the interface between 2 flowing streams of solutions of different pCa. Data were collected and analyzed using customized LabView software. Measured mechanical and kinetic parameters were defined as follows: resting tension (mN/mm^2^) = myofibril basal tension in fully relaxing condition; maximal tension (mN/mm^2^) = maximal tension generated at full calcium activation (pCa 4.5); the rate constant of tension development following maximal calcium activation = *k*_ACT_; duration of the linear relaxation = linear duration; and the rate constant of exponential relaxation = fast *k*_REL_. For myofibril resting tension–to–sarcomere length curves, each myofibril was stretched incrementally to determine the resting tension at different sarcomere lengths, and a resting tension–sarcomere length relationship was measured. The data from all myofibrils of the same animal were grouped into 0.15-μm intervals of sarcomere length and averaged. The averaged data of all animals from each group were shown as mean ± (or +) SEM values fitted by third-order polynomials. At least 1 sample from each treatment group or genetic background was analyzed on a given day. All cultured cells in a particular experiment were harvested on the same day. While myofibril experiments were conducted by the person preparing the samples and thus were not blinded to genotype or drug treatment, the data were analyzed in a blinded fashion.

### Ex vivo treatment of myofibrillar proteins with HDAC6 and/or PKCα.

Myofibril pellets were washed twice with reaction buffer (25 mM Tris-HCl, pH 8.0, 137 mM NaCl, 2.7 mM KCl, 1 mM MgCl_2_, 0.1 mg/mL BSA). Washed myofibril lysates were resuspended in 200 μL reaction buffer with or without recombinant HDAC6 (1 μg/μL; BPS Bioscience) for 30 minutes at 15°C. For the treatment of recombinant PKCα and recombinant HDAC6, myofibril lysates were resuspended in 150 μL of relaxing solution containing recombinant PKCα (0.066 U/μL; Millipore 14-484) and lipid activator (Millipore 20-133) for 30 minutes with or without the addition of recombinant HDAC6 (1 μg/μL; BPS Bioscience) for another 30 minutes. After treatment, myofibril lysates were washed twice with relaxing solution containing protease inhibitors, and mechanics studies were performed. Homogenized myofibrils were centrifuged at 13,000*g* for 5 minutes, then separated using agarose gels.

### Statistics.

Mean ± SEM values are shown and were compared by 2-tailed Student’s *t* test (2 unpaired groups), 1-way ANOVA (>2 groups), or mixed-effects models for repeated measures followed by Tukey’s or Bonferroni’s multiple-comparison tests where appropriate. A *P* value less than 0.05 was considered statistically significant.

### Study approval.

Animal experiments were reviewed and approved by the Institutional Animal Care and Use Committee at the University of Colorado Anschutz Medical Campus. Human hearts were obtained from a tissue bank, which was reviewed and approved by the Colorado Multiple Institutional Review Board (COMIRB 01-568) and is maintained by the Division of Cardiology at the University of Colorado Anschutz Medical Campus. Non-failing human hearts were obtained from individuals who provided written informed consent to be donors. 

## Author contributions

YHL, MYJ, KCW, and TAM conceived and designed the study. YHL, JLM, TL, JGT, SAW, KRH, MAC, ZH, CEW, and KCW acquired data. YHL, JLM, TL, SAW, KRH, ZH, YH, MG, SKF, MPYL, CC, HLG, KCW, and TAM analyzed and interpreted data. YHL, JLM, JGT, MG, MPYL, HLG, KCW, and TAM wrote and reviewed the manuscript. MYJ, SAW, KRH, MAC, CEW, and AVA provided administrative, technical, or material support. MYJ, HLG, KCW, and TAM supervised the study. The order of the first or last authors reflects the leadership exerted in the study.

## Supplementary Material

Supplemental data

## Version 1 05/16/2022

Electronic publication
