# Supplementary material for: HDAC6 modulates myofibril stiffness and diastolic function of the heart
Source: J Clin Invest. 2022 May 16;132(10):e148333. doi: 10.1172/JCI148333 (PMC9106344; doi:10.1172/JCI148333)

Supplemental Material

Expanded Material and Methods

Animal models

Sprague Dawley (SD) rats were obtained from Charles River Laboratories for ARVM isolation. For the hypertension-induced DD model, 10-week-old male WT and HDAC6 KO mice, backcrossed to the sv129 S6/SvEvTac background, were subjected to right unilateral nephrectomy (UNX). One week later, mice underwent a subcutaneous implantation of 25mg deoxycorticosterone acetate (DOCA; Sigma Aldrich D7000) impregnated in a medical grade 100% silastic pellet (approximately 1000mg/kg) or a sham-vehicle pellet. Following implantation, mice that received the DOCA pellet were given tap water containing 0.9% NaCl and 0.2% KCl to drink. The aging study was conducted with male WT and HDAC6 KO mice on the sv129 S6/SvEvTac background. For evaluation of PEVK KO titin, myofibrils were obtained from frozen LVs of 4-7-month-old male WT and KO mice on a mixed C57BL/6 x 129S6 background and from the same colony.

Hemodynamic analysis

Serial transthoracic echocardiography and Doppler analyses were performed using a Vevo2100 instrument (VisualSonics) to simultaneously obtain ECG recordings and assess cardiac dimensions and LV function. Animals were anaesthetized with 2% isoflurane, their chests shaved, and body temperature maintained at 37^0C. Long and short parasternal axes views of the LV were obtained. Short axis 2-dimensional views of the LV at the papillary muscle level were used to acquire M-mode targeted recordings. Anterior and posterior end-diastolic and end-systolic LV wall thickness and internal diameter were measured using the leading edge method of the American Society of Echocardiography to calculate ejection fraction (EF). Doppler signals

of mitral inflow and myocardial tissue movement at the level of the mitral annulus were obtained to calculate the ratio of early and active filling wave-peak of diastolic flow velocity (E/A), and the ratio of peak diastolic tissue velocity (E'/A'), in order to assess diastolic cardiac function. All measurements were averaged from three consecutive cardiac cycles on the exhale phase. All echocardiographic measurements and analyses were performed in a blinded manner by a dedicated small animal echocardiography and hemodynamics team. Serial blood pressure measurements were taken in conscious animals using a noninvasive tail-cuff system (CODA, Kent Scientific). To minimize the impact of anxiety on blood pressure readings, mice were taken through the entire procedure for 5 consecutive days prior to obtaining the measurements. Immediately prior to the conclusion of the study, LV end diastolic pressure was measured using an invasive catheter in mice anesthetized with 1.5% isoflurane (Scisense, Inc.).

Exercise testing

Exercise tests were performed on a custom-built motor-driven treadmill (Exer 3/6 Treadmill, Columbus Instruments, Columbus OH, USA) following a 5-day acclimatization period in which animals were familiarized with treadmill running for 5 minutes/day at a speed of 10m/min up a 5% incline. For the last several runs, the speed of the treadmill was increased progressively over the last minute to ~15-25 m/min to familiarize the mice with high-speed running. Following a brief 2-minute run at 10m/min and a 1-minute rest (warm-up), mice underwent a series of 4 runs to exhaustion at speeds of 10, 20, 30, and 40 m/min performed at random on separate days for the determination of critical speed (CS). Mice were encouraged to run by applying manual bursts of high-pressure air at the hindlimbs whenever they drifted towards the back of the treadmill lane. Tests were terminated whenever the mice began to show an obvious change in running gait, remained at the rear of the treadmill, or were unable or unwilling to keep pace with the treadmill belt despite obvious exertion of effort. CS was calculated from the slope (a)

and the intercept (b) of the regression line, plotting the distance vs. the time to exhaustion from the 4 tests according to the equation y=ax+b, as previously described (1).

Tissue procurement

After hemodynamic recordings, animals were sacrificed by exsanguination and hearts excised and placed in ice-cold saline. RV was dissected from LV by cutting along the septum and the outer wall of the LV, and all parts were weighed. Fifty milligram biopsies of the LV were flash-frozen in liquid nitrogen for subsequent biochemical and myofibril mechanics analyses. A remaining portion of the LV was mixed with optimal cutting temperature (OCT) compound prior to freezing for subsequent histological assessment. Tibias were cleaned and lengths were measured.

Histological analysis

Frozen tissue sections were cut at a thickness of 5 µm. For fibrosis analysis, cardiac sections were stained with Picrosirius red (PSR) dye for 1 hour. Fifteen randomly chosen images were taken for each section using the Keyence BZ-X710 All-in-One Fluorescence Microscope. Quantification of PSR staining was completed by determining the ratio of positively stained (red) pixels to the total pixel number of each image (% fibrosis) using Axiovision Software. The mean % fibrosis for each section (from all 15 images) was calculated.

Cardiomyocyte culture

Adult rat left ventricular myocytes (ARVMs) were cultured from female Sprague Dawley rats (250-300 grams) as described (2). Briefly, the heart was removed and retrograde perfused (120.5 mM NaCl, 14.7 mM KCl, 0.6mM KH_2PO_4, 0.6mM Na2HPO$_4$, 1.2mM $MgSO_4$, 4.6 mM $NaHCO_3$, 10 mM

Na-HEPES, 30 mM Taurine, 10 mM 2,3-butanedione monoxime, 5.5 mM Glucose, pH 7.2) for 10 minutes at 37°C, then digested with Liberase DH (Roche, 0.33 mg/mL) for 8 minutes. The heart was then minced and the slurry filtered through sterile 150-nm mesh. Myocytes were separated by centrifugation at 400g for 4 minutes and the myocyte suspension was layered over 60 μg/mL of BSA and allowed to settle for 15 minutes. Myocytes were plated on 100-mm laminin-coated plastic culture dishes at a density of 100 to 150 cells/mm^2. ARVM culture was maintained in serum-free DMEM supplemented with albumin (2 mg/ml), carnitine (2 mmol/l), creatine (5 mmol/l), taurine (5 mmol/l), BDM (1 mg/ml), and penicillin-streptomycin (100 μg/ml). For HDAC inhibitor treatment, ARVMs were exposed to ITF2357 (1 μM), tubastatin A (10 μm) or DMSO (0.1% final concentration) for 24 hours.

For isolation and culture of adult mouse ventricular myocytes (AMVMs), cells were obtained from 3 female PEVK KO and WT littermate controls between 5 and 6 months of age as described previously (3). AMVMs were cultured in the presence of tubastatin A (10 μm) or DMSO (0.1% final concentration) for 24 hours.

Indirect immunofluorescence

ARVMs were fixed with 4% paraformaldehyde at room temperature for 30 minutes. Cells were permeablized with PBS-T (PBS containing 0.3% Triton-X-100) for 30 minutes and then blocked with 10% fetal bovine serum (Gemini Bio-Products) in PBS-T for 30 minutes at room temperature. Following blocking, cells were incubated in primary antibodies overnight (mouse α-tubulin (1:200; Santa Cruz Biotechnology, sc-23948); mouse acetyl-α-tubulin (1:200; Santa Cruz Biotechnology, sc-23950); rabbit DYKDDDDK (FLAG) tag (Cell Signaling Technology, #14793); mouse α-actinin (1:1000; Sigma, A7811)). Secondary antibodies (Invitrogen, Goat anti-Mouse IgG (H+L) Alexa Fluor 555, A21422, 1:1000; Goat anti-Rabbit IgG (H+L) Alexa Fluor 488, A11034, 1:1000) were applied for 3 hours at room temperature. Coverslips were mounted on glass slides using mounting

medium (Vector Laboratories, H-1200). Confocal images were obtained using an Olympus FLUOVIEW FV1000 confocal laser scanning microscope (UC Denver, Advanced Light Microscopy Core).

SDS-PAGE and immunoblotting

ARVMs were washed in PBS twice then collected in isoelectric focusing buffer (IEF; 8M urea, 2.5M thiourea, 4% CHAPS, 2mM EDTA, 1mM DTT, 1% TBP, phosphatase and protease inhibitors (Sigma Aldrich)). Twenty micrograms of protein was separated by SDS-PAGE on a 12% gel and transferred to PVDF membrane (Bio-Rad, 162-0177). The membranes were blocked with 5% milk in TBS for 30 minutes then incubated in primary antibodies overnight at 4°C (mouse α-tubulin (1:1000; Santa Cruz Biotechnology, sc-23948); mouse Acetyl-α-tubulin (1:1000; Santa Cruz Biotechnology, sc-23950); mouse ANTI-FLAG® M2 antibody (1:2000; Simga, F1804); mouse TTN monoclonal Z1Z2 antibody (1:2500; Abnova, H00007273-M06), rabbit phospho-TTN S12022 antibody (1:2000; Genescript, custom-made), rabbit phospho-TTN S11878 antibody (1:2000; GL Biochem, custom-made) and rabbit phospho-TTN S4010 antibody (1:500; GL Biochem, custom-made). Membranes were washed 3 times in TBS with 1% tween 20 and then incubated in goat-anti-mouse secondary antibodies (1:2000, SouthernBiotech). Protein was quantified using ImageJ and acetylation signal of α-tubulin was normalized to total α-tubulin level for each sample. Titin protein was isolated in solubilization buffer (8M Urea, 2M Thiourea, 3% sodium dodecyl sulfate, 75mM DTT, 50mM Tris-HCl, 0.03% bromophenol blue) and separated using SDS-agarose gel electrophoresis.

Mass spectrometry sample preparation

LVs of 6-month-old male WT and HDAC6 KO mice were mixed with SDC lysis buffer individually (2% Sodium deoxycholate, 50 mM Hepes, pH 8.0). Tissue was lysed in a tissue homogeniser

(Bertin Precellys Evolution (6800rpm, 12x20 s, 30 s pause, 0°C)). The lysates of knockout and wild type samples were pooled respectively. Lysates were boiled at 95°C for 10 min and protein concentration was determined by BCA assay (Pierce). 70 mg of knockout and wild type sample were mixed with 5 mM Tris(2-carboxyethyl)phosphine and 5.5 mM chloroacetamide to reduce and alkylate disulphide bonds (1h, 30°C). Proteins were digested into peptides over night with Trypsin (1:100 w/w, Promega) at 37°C. Digestion was terminated by adding trifluoroacetic acid (TFA) to a final concentration of 3%. Peptide mixture was cleared by centrifugation (3,800xg, 20 min) and loaded onto C18 reverse phase Sep-Pak classic cartridges (Waters). Columns were washed with 10 ml 0.15% TFA two times and dimethyl labelled, as previously described (4). HDAC6 knockout peptides were labelled with the light dimethyl reagent (5x7ml; 11.11 mM Monosodium phosphate, 38.89 mM Disodium phosphate, 0.04% formaldehyde, 6 mM cyanborohydride, pH 7.5) resulting in a mass shift of 28 Da per terminal amine. Peptides from the wild type sample were labelled with the intermediate reagent (11.11 mM Monosodium phosphate, 38.89 mM Disodium phosphate, 0.04% deuterated formaldehyde, 6 mM cyanborohydride, pH 7.5) resulting in a mass shift of 32 Da per terminal amine. Briefly, peptides bound to Sep-Pak columns were washed five times with 7 ml of the corresponding labelling reagent followed by a wash step with 10 ml 0.15% TFA and elution in 3 ml 50% Acetonitrile (ACN) per column. The labelling efficiency for the HDAC6 knockout sample was 98.5% and for the wild type sample 99.3%. Acetylated peptides were enriched with the PTMscan acetyllysine antibody kit (Cell Signaling Technology) followed by in-tip SCX purification, in-tip high pH fractionation (10 fractions, 5-50% ACN) and a final desalting by in-tip C18 reverse-phase chromatography, as previously described (5). A proteome measurement was prepared by purifying peptides by in-tip SCX purification followed by high pH fractionation (10 fractions, 5-50% ACN) and in-tip C18 desalting. Each fraction was vacuum dried and peptides were re-dissolved in MS buffer (0.1% Formic acid) prior to measurement.

Mass spectrometry

Fractionated peptides were analyzed by online liquid chromatography coupled tandem mass spectrometry utilizing a Proxeon EASY-nLC1200 (Thermo Scientific) attached to an Exploris480 (Thermo Scientific). Peptide fractions were loaded onto a chromatography column (length 15 cm, inner diameter 75 µm) packed with C18 reverse-phase material (Reprosil-Pur Basic C18, 1.9 µm, Dr. Maisch GmbH) and eluted by a 75 min gradient for enriched samples and 145 min gradient for proteome samples (5-40% ACN/H2O and 0.1% formic acid). Eluting peptides were ionized by electro spray ionization and injected into the mass spectrometer under the specified settings: 2.3 kV spray voltage, no sheath/aux/sweep gas flow rate, 270°C capillary temperature, and funnel RF at 40%. The Exploris480 was operated by Xcalibur (v. 4.4.16.14) and Orbitrap Exploris 480 Tune (v. 2.0.182.25) in data dependent mode. MS1 scans were conducted at a resolution of 120,000 (200 m/z), in Top12 mode and a scan range of 300-1,750 m/z. The normalized AGC target for the scans was 300% and maximum injection time 60 ms. Peptides were fragmented at a normalized collision energy of 25 by Higher-energy collisional dissociation (HCD) before MS2 measurement. MS2 scans were performed at a resolution of 15,000 (200 m/z), an normalized AGC target of 80% and a maximum injection time of 110 ms, isolation window of 1.3 m/z and a fixed first mass of 100 m/z. The dynamic exclusion was set to 30 s and ions with a charge state between two and five were included to the analysis.

Raw data were processed with MaxQuant (2.0.1.0) by a search against the Mus musculus UniProt database (downloaded May 3, 2021) and quantification of the Dimethyl pairs for light (DimethLys0, DimethNter0) and intermediate (DimethLys4, DimethNter4). The false discovery rate was kept at 1% for peptides and proteins. Cysteine carbamidomethylation was set as a fixed modification and Methionine oxidation, N-terminal acetylation, Lysine acetylation and deamination of Glutamine and Asparagine was set as variable. Known contaminants and reverse peptides from the target decoy approach were removed from the results as well as Acetylation sites with a localisation probability of <0.9.

Supplemental References

1. Billat VL, Mouisel E, Roblot N, and Melki J. Inter- and intrastrain variation in mouse critical running speed. *J Appl Physiol (1985).* 2005;98(4):1258-63.
2. Woulfe KC, Ferrara C, Pioner JM, Mahaffey JH, Coppini R, Scellini B, et al. A Novel Method of Isolating Myofibrils From Primary Cardiomyocyte Culture Suitable for Myofibril Mechanical Study. *Front Cardiovasc Med.* 2019;6:12.
3. Travers JG, Wennersten SA, Pena B, Bagchi RA, Smith HE, Hirsch RA, et al. HDAC Inhibition Reverses Preexisting Diastolic Dysfunction and Blocks Covert Extracellular Matrix Remodeling. *Circulation.* 2021;143(19):1874-90.
4. Hsu JL, Huang SY, Chow NH, and Chen SH. Stable-isotope dimethyl labeling for quantitative proteomics. *Anal Chem.* 2003;75(24):6843-52.
5. Hansen BK, Gupta R, Baldus L, Lyon D, Narita T, Lammers M, et al. Analysis of human acetylation stoichiometry defines mechanistic constraints on protein regulation. *Nat Commun.* 2019;10(1):1055.

Supplemental Fig. 1

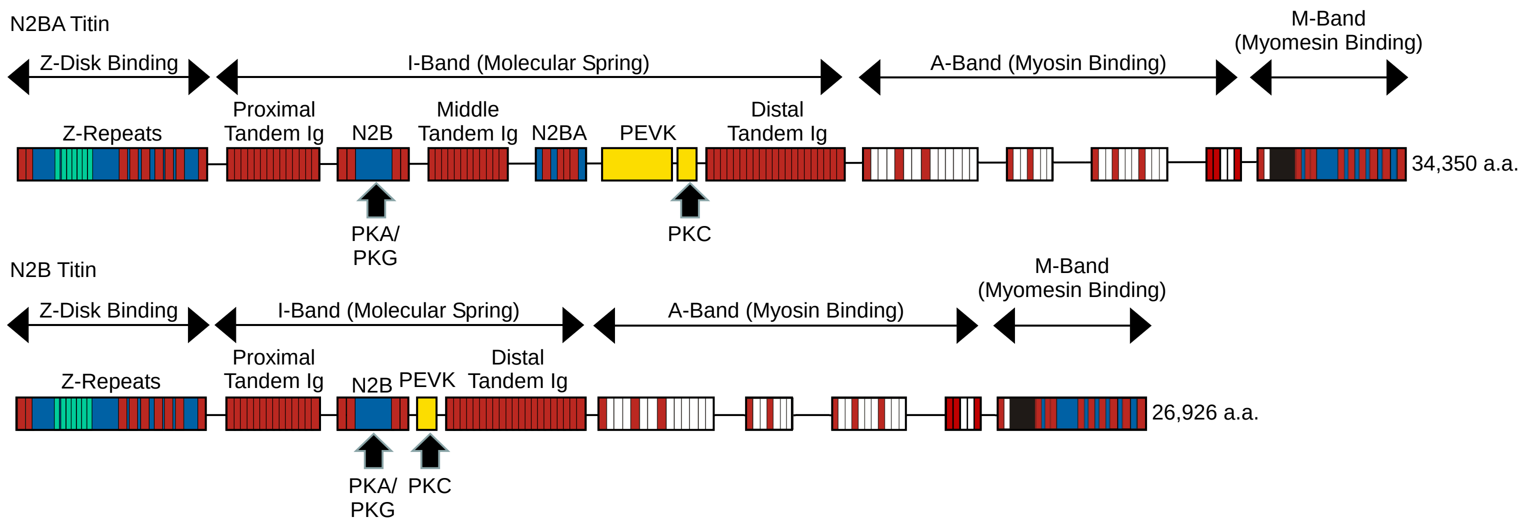

Supplemental Fig. 1. Schematic representations of titin. Titin is a modular protein that is initially expressed as a fetal isoform, N2BA, which is subject to splicing by RBM20 to yield the adult isoform, N2B. Both isoforms consist of an amino-terminal Z-disk-binding region followed by an I-band spring region, which is the primary location of differential splicing. Both titin isoforms harbor a common N2B element within the spring region. However, the spring region of the N2BA isoform contains more immunoglobulin-like (Ig) domain segments and a longer PEVK element, so named because it is rich in proline, glutamic acid, valine and lysine residue. As such, with an extended spring region, the N2BA isoform is more compliant than the adult N2B isoform of titin. Other domains that are common to both isoforms of titin comprise the myosin-binding A-band, and the M-band, which binds myomesin. Sites of phosphorylation by protein kinase A (PKA), protein kinase G (PKG) and protein kinase C (PKC) are indicated.

Supplemental Fig. 2

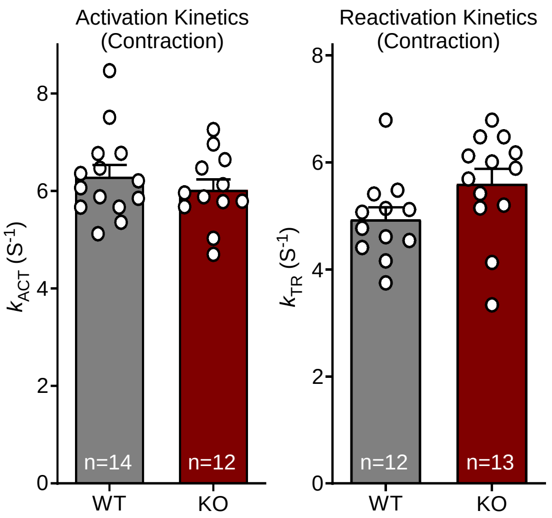

Supplemental Fig. 2. HDAC6 deletion does not alter the kinetics of cardiac myofibril contraction. Kinetic parameters of myofibril tension generation (k_{ACT}, rate constant of tension generation following Ca^{2+} activation; and k_{TR}, rate constant of tension redevelopment following release-restretch) were unaffected by HDAC6 deletion.

Supplemental Fig. 3

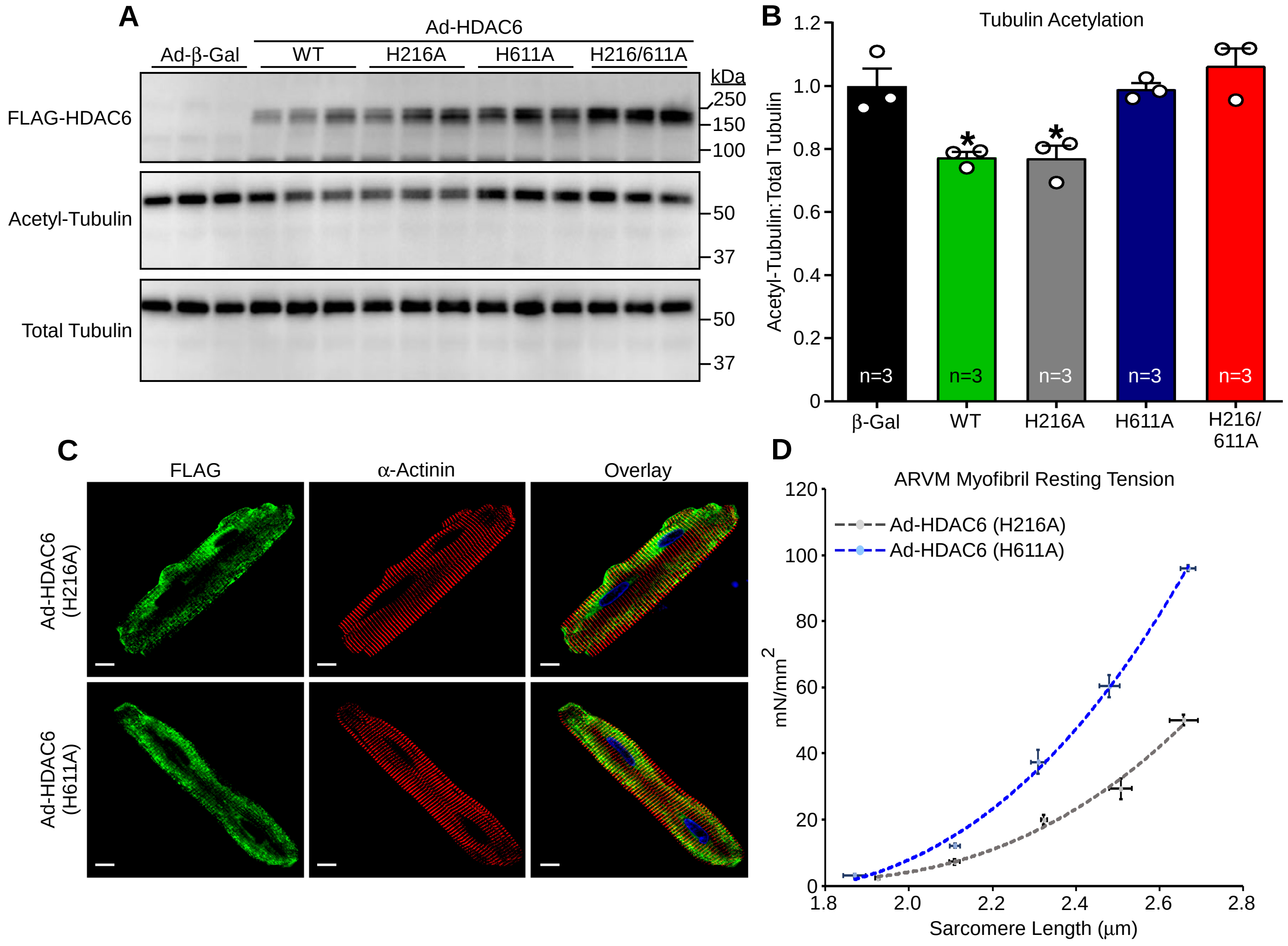

Supplemental Fig. 3. The activity of deacetylase domain 2 of HDAC6 is required to reduce cardiac myofibril stiffness. (A) Adult rat ventricular myocytes (ARVMs) were infected with the indicated adenoviruses and whole-cell homogenates were immunoblotted with antibodies against the FLAG epitope, acetyl-tubulin and total tubulin. (**B**) Densitometry quantification of the immunoblot signals in **A**. Mean ±SEM values are shown and were compared by two-way ANOVA with Tukey's multiple comparisons test. *$P<0.05$ vs β-Gal control. (**C**) Confocal, indirect immunofluorescence of ARVMs infected with adenoviruses encoding FLAG-tagged versions of HDAC6 harboring an amino acid substitution that abolishes the catalytic activity of deacetylase domain 1 (H216A) or deacetylase domain 2 (H611A). Scale bar = 10 μm. (**D**) Myofibril resting tension-to-sarcomere length curves. 6-8 myofibrils per animal, 4 animals per group were analyzed.

Supplemental Fig. 4

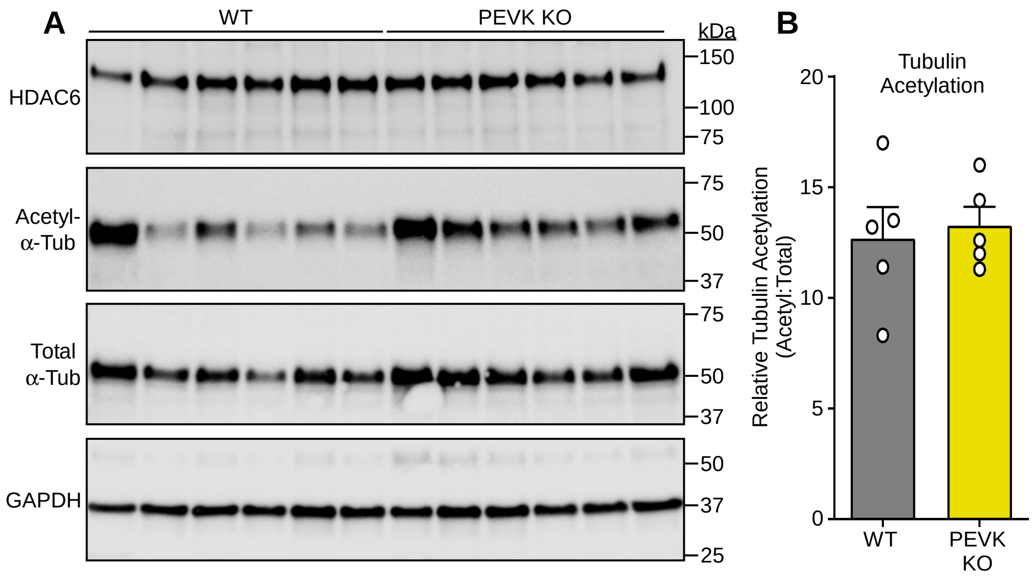

Supplemental Fig. 4. HDAC6 expression and levels of tubulin acetylation are not altered by deletion of the PEVK element in titin. (A) LV homogenates were immunoblotted with the indicated antibodies, with each lane representing protein from an independent mouse. (**B**) Densitometry quantification revealed no differences in the signals between WT and PEVK KO hearts.

Supplemental Fig. 5

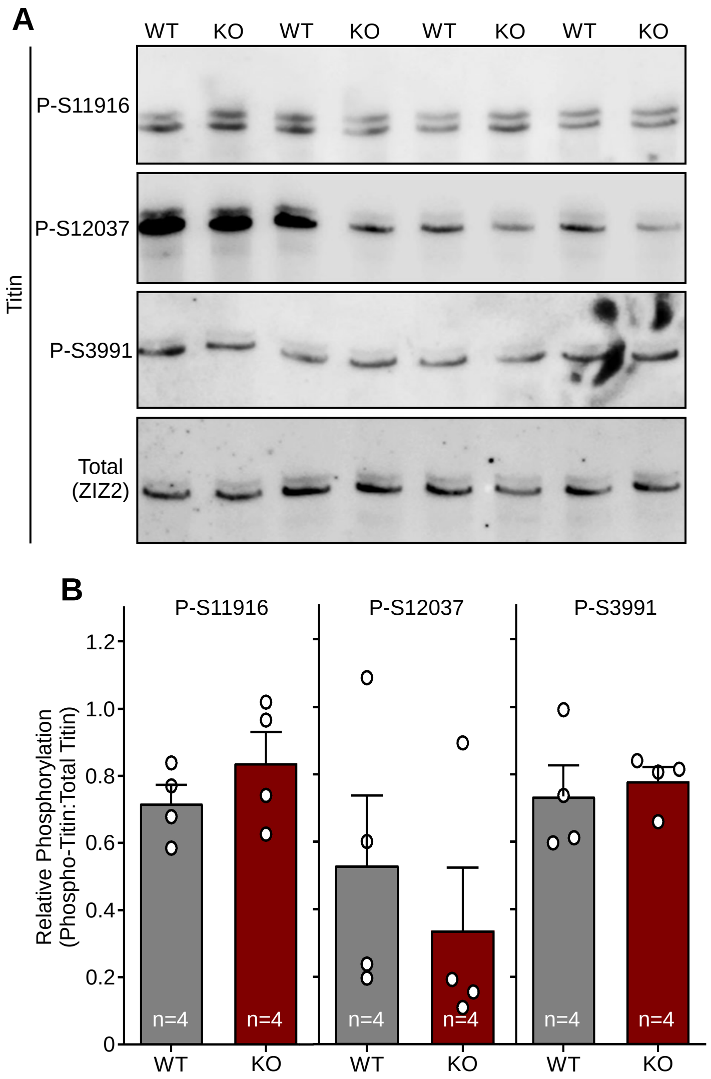

Supplemental Fig. 5. HDAC6 deletion does not alter site-specific phosphorylation of the N2B or PEVK regions of titin. (**A**) Myofibrillar proteins were prepared from left ventricular homogenates from WT and HDAC6 KO mice. Titin was resolved by SDS-PAGE and immunoblotted with antibodies specific for phospho-S11916 (S11878 in human) and phospho-S12037 (S12022 in human) in the PEVK domain, phospho-S3991 (S4010 in human) in the N2B region, as well as an antibody to the titin Z1Z2 element to asses total titin levels. (**B**) Densitometry of immunoblots was used to quantify site-specific titin phosphorylation relative to total titin. Total titin was normalized to total myosin heavy chain (MyHC) levels determined by Coomassie Brilliant Blue gel staining.

Supplemental Fig. 6

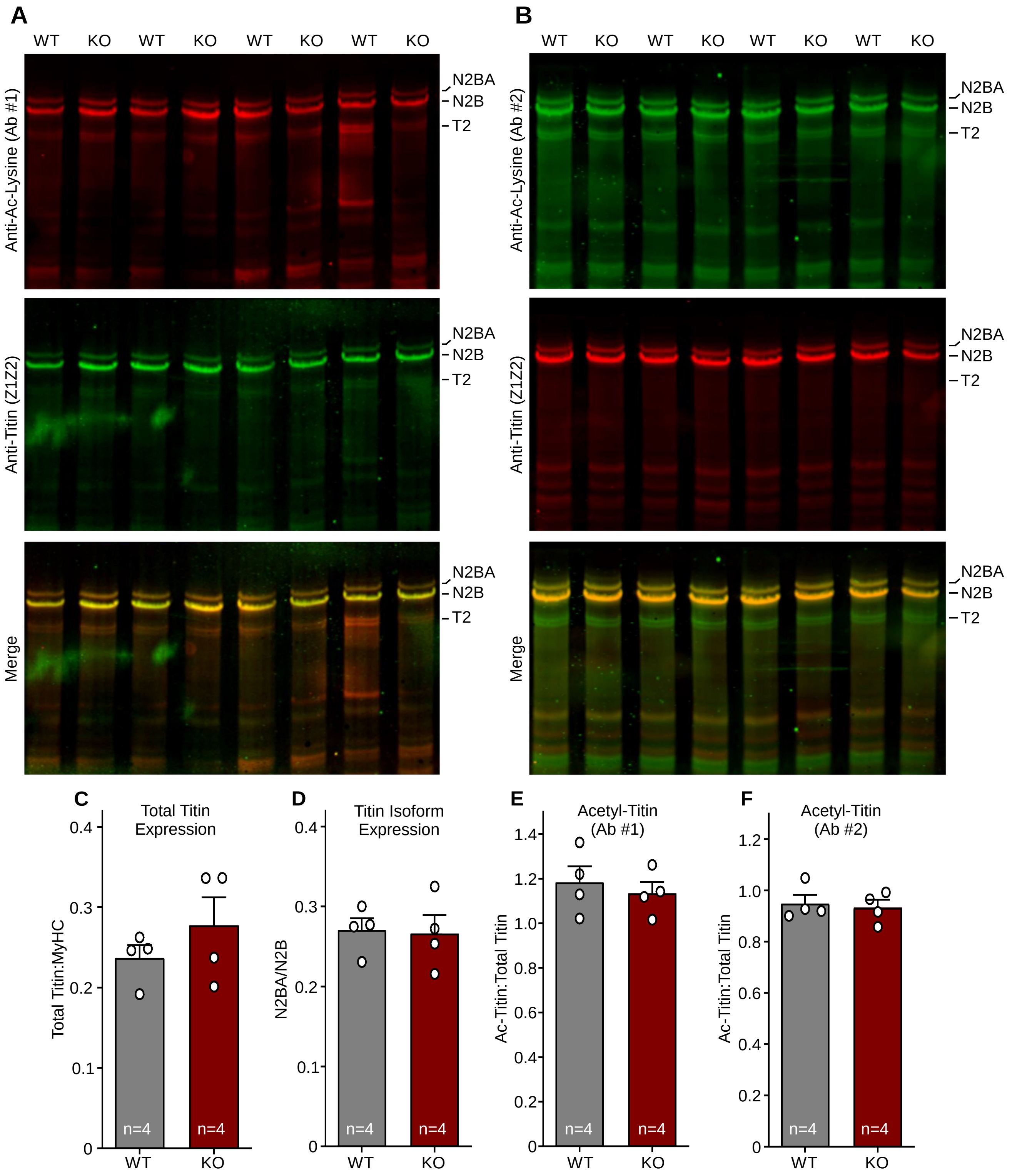

Supplemental Fig. 6. HDAC6 deletion does not alter titin isoform levels or global titin acetylation. (A and **B)** Myofibrillar proteins were prepared from left ventricular homogenates from WT and HDAC6 KO mice. Titin was resolved by SDS-PAGE and immunoblotted with two distinct antibodies against acetyl-lysine as well as an antibody to the titin Z1Z2 element to assess total titin levels. (**C** to **F**) Densitometry of immunoblots was used to quantify acetylation of titin, relative expression of titin N2BA and N2B isoforms, and total titin. T2 represents a cleavage product of titin. Total titin was normalized to total myosin heavy chain (MyHC) levels determined by Coomassie Brilliant Blue gel staining.

Supplemental Fig. 7

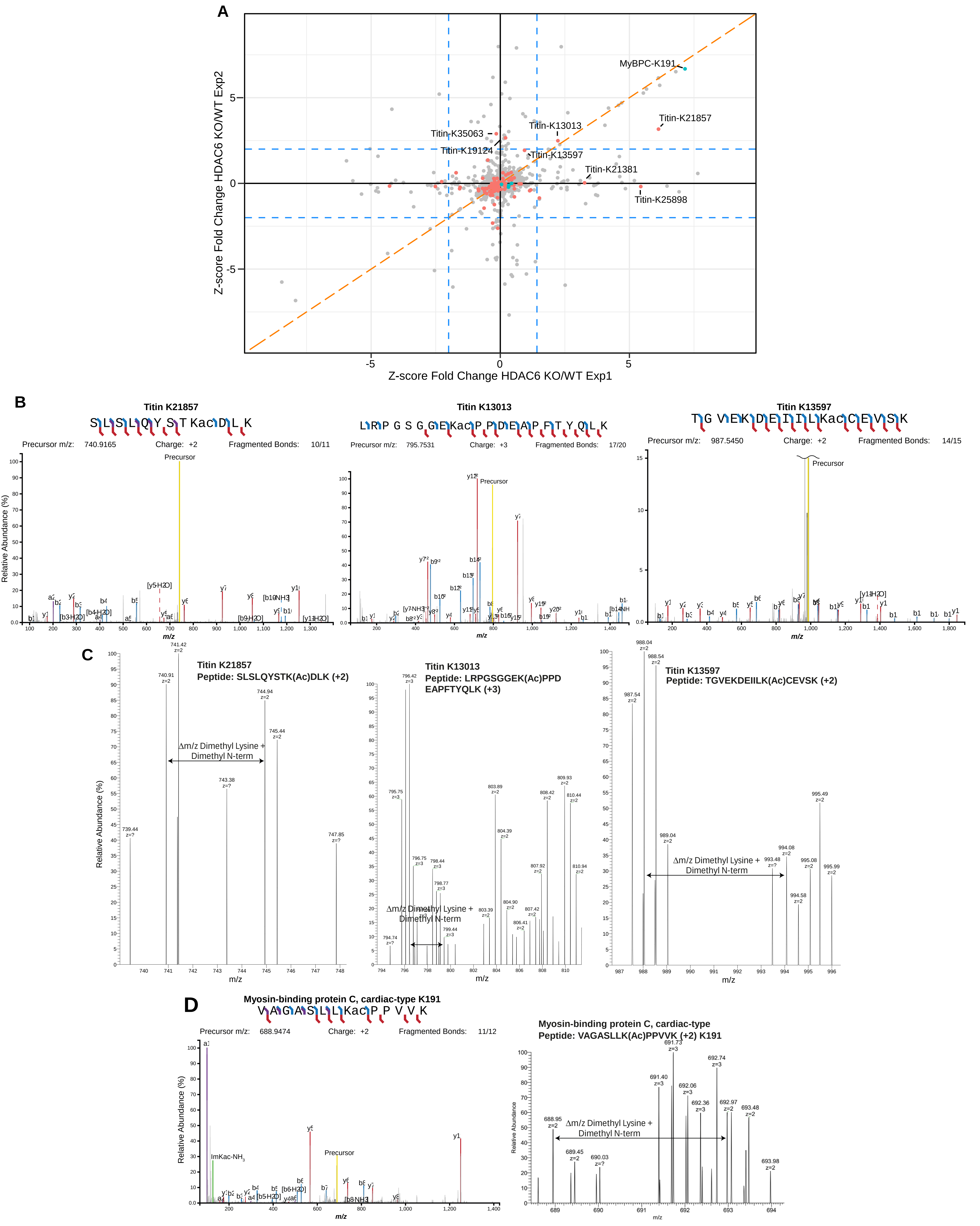

Supplemental Fig. 7. Site-specific alterations in protein acetylation in hearts of HDAC6 KO mice. (A) Mass spectrometry-based acetylproteomics analysis was performed with LV lysates to address the impact of HDAC6 deletion on site-specific lysine acetylation in the heart. Online liquid chromatography coupled tandem mass spectrometry of affinity-purified acetylated peptides derived from trypsin digested total LV protein homogenates revealed an increase in acetylation of several lysines in titin in HDAC6 KO hearts, including two sites adjacent to the PEVK element (K13013 and K13597). Acetylation of K-191 of cardiac myosin-binding protein C (MyBP-C) was also increased in HDAC6 KO hearts. (**B** and **C**) Representative titin precursor spectra are shown, and annotated MS2 spectra confirmed the identity of specific lysine acetylation sites. (**D**) Precursor mass spectrum and annotated MS2 fragment spectrum of the peptide harboring K-191 of MyBP-C are shown.

Supplemental Fig. 8

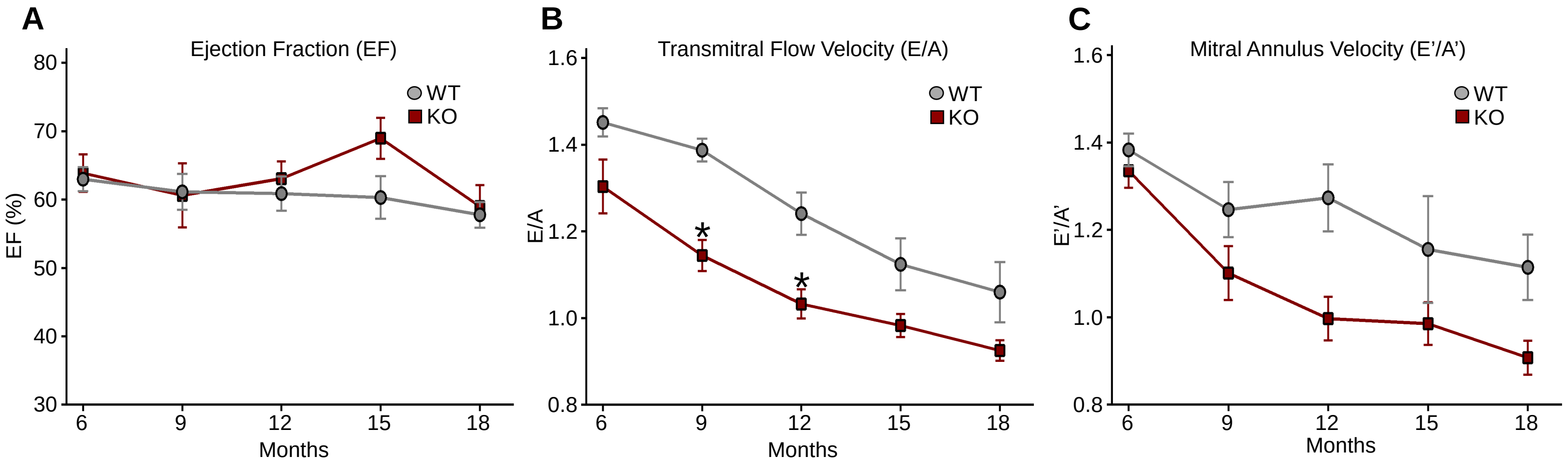

Supplemental Fig. 8. Evidence of exaggerated age-dependent diastolic dysfunction in HDAC6 KO mice. Serial echocardiographic assessment of ejection fraction (EF) (**A**), mitral inflow velocities (E/A) (**B**), and septal mitral annulus velocities (E'/A') (**C**) are shown. The data are presented as mean +/-SEM, with statistical analysis performed using a mixed-effects model for repeated measures with Bonferroni's multiple comparisons test. *$P<0.05$ vs. WT at the same age. Mouse numbers for each time point are provided in Supplemental Table 1, and additional echocardiographic data are provided in Supplemental Table 3.

Fig. 2D acetyl-tubulin

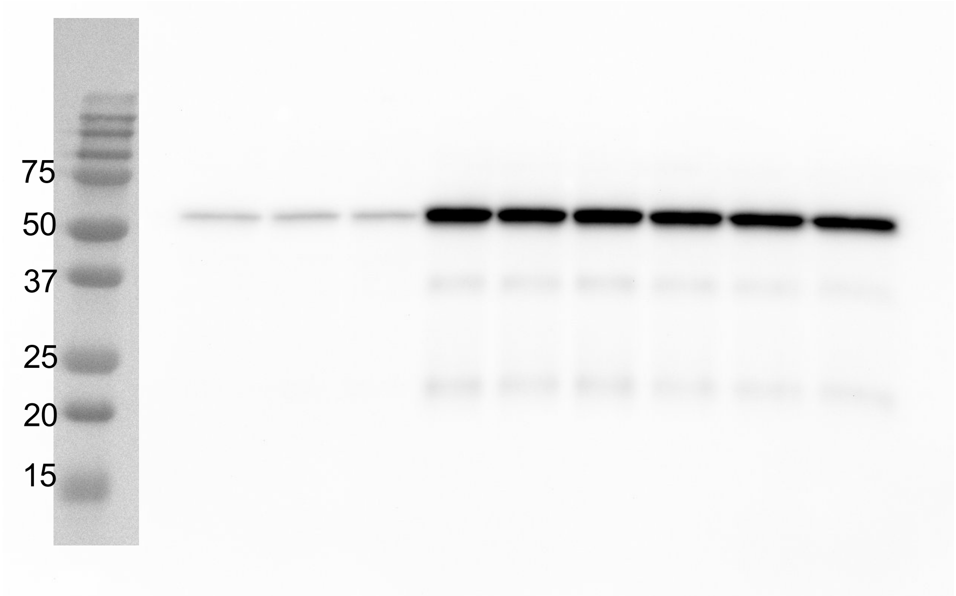

Fig. 2D total tubulin

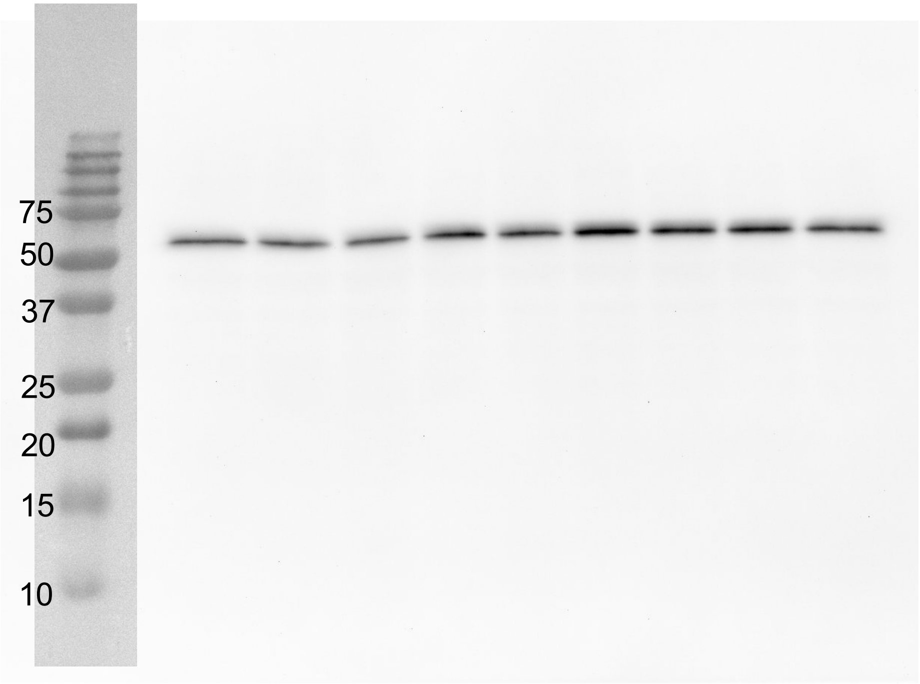

Fig. 5E – phospho-PKC sites

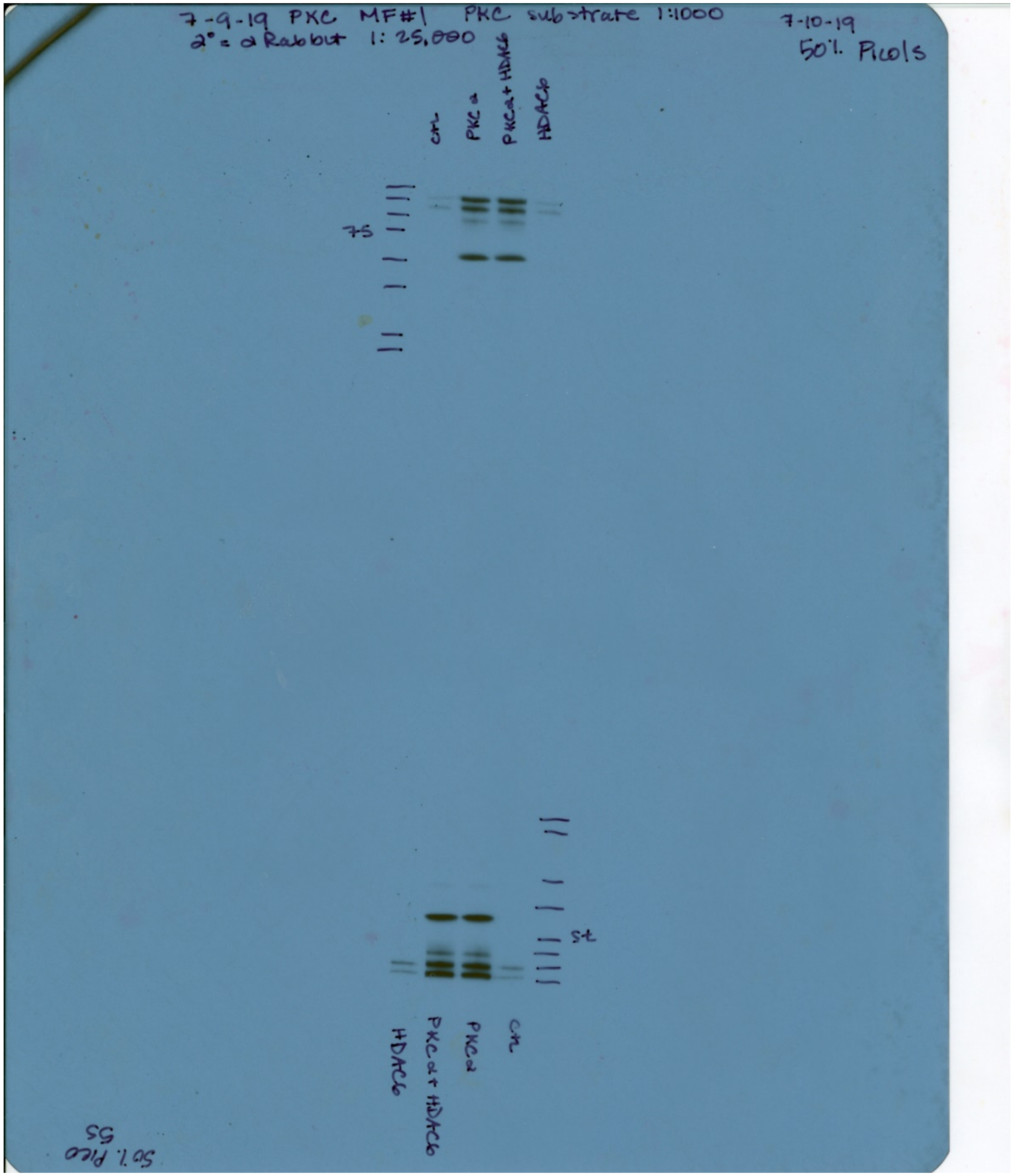

Fig. 5F – pS26 = p11916

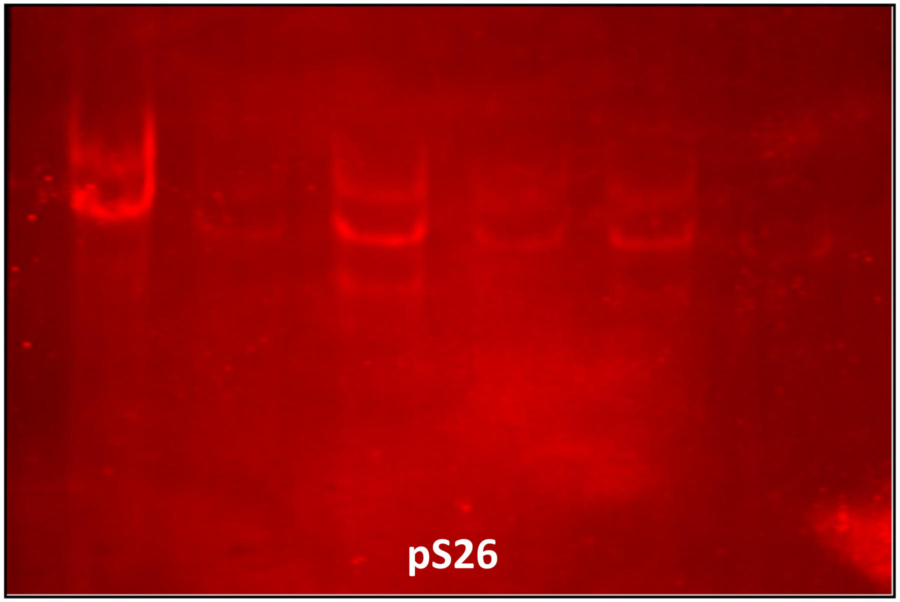

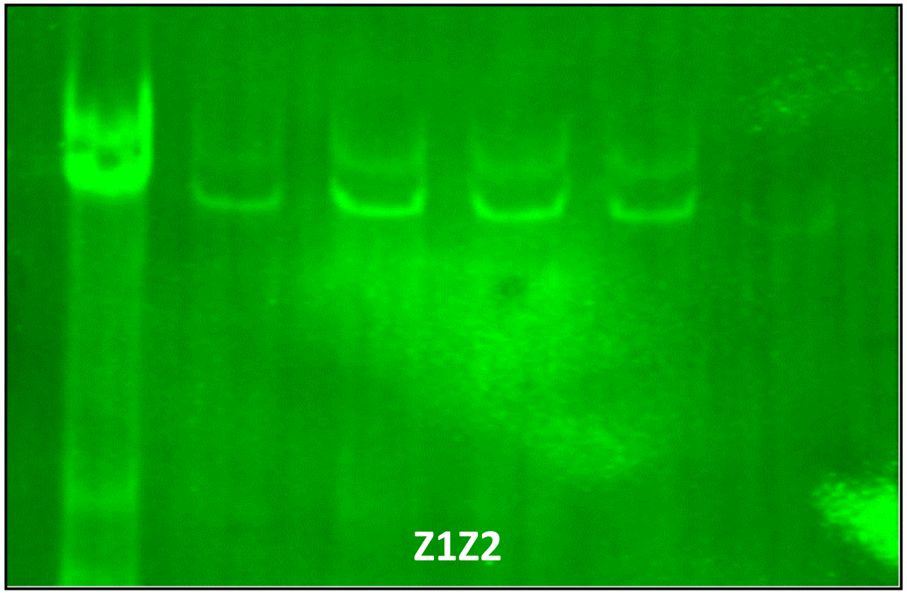

Fig. 5F – pS170 = p12037

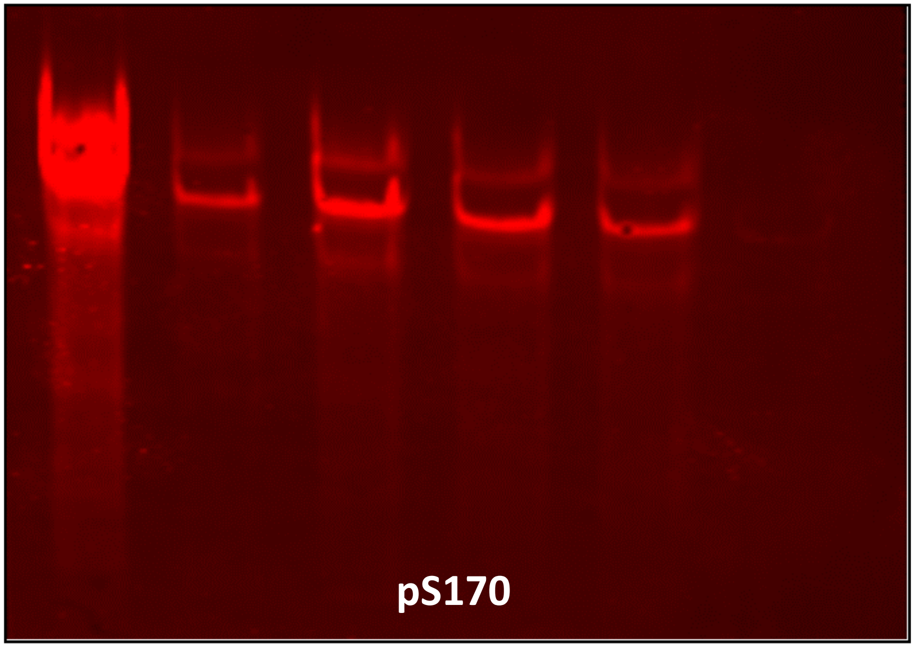

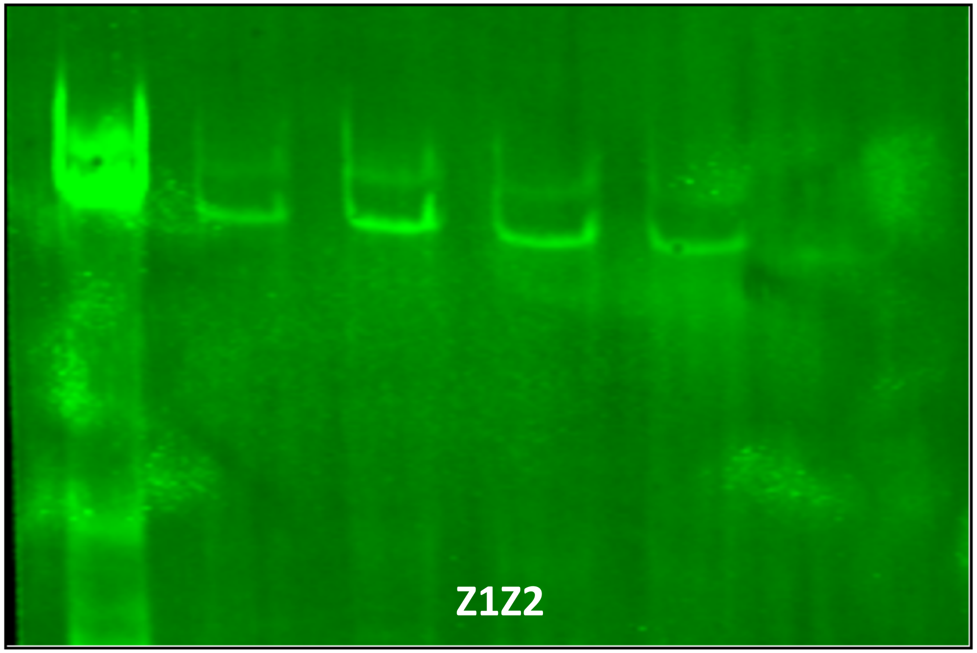

sFig. 3 – HDAC6 (membrane was cut in half to probe for HDAC6 and tubulin separately)

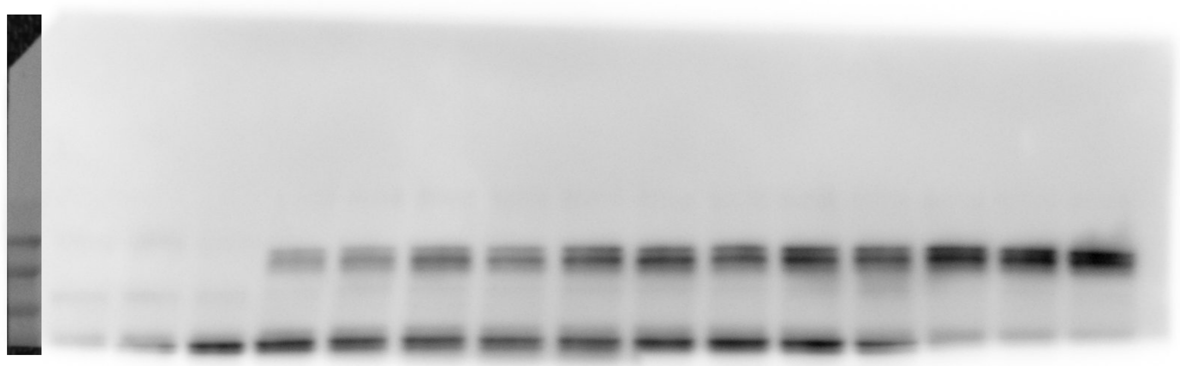

sFig. 3 – Total Tubulin (membrane was cut in half to probe for tubulin and HDAC6 separately)

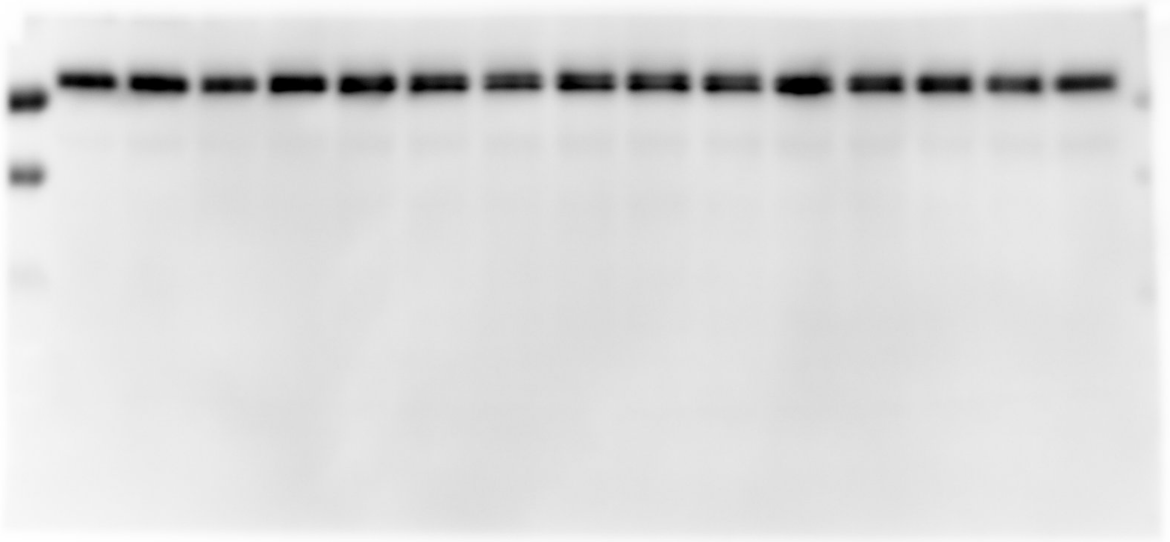

sFig. 3 – Acetyl-Tubulin (membrane was cut in half to probe for ac-tubulin and HDAC6 separately)

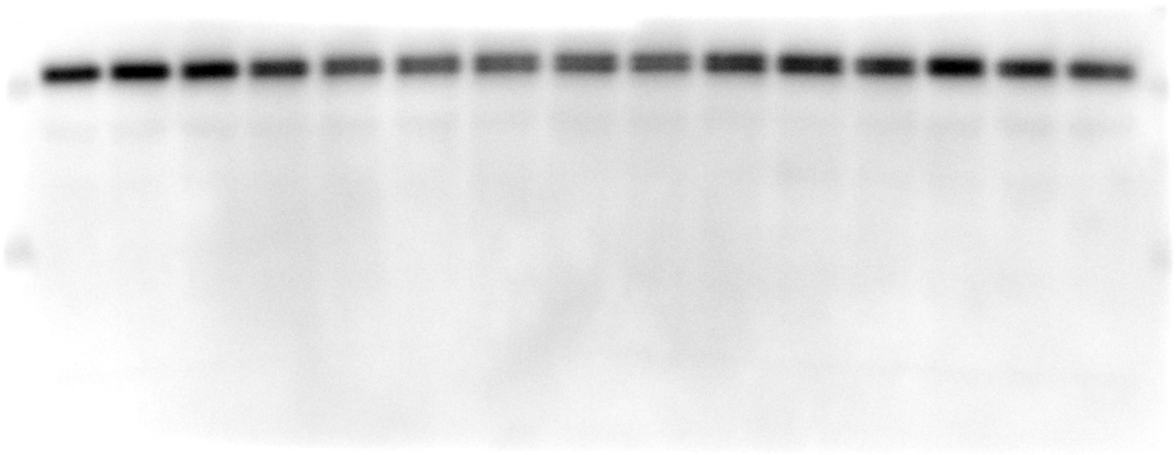

sFig. 4 – HDAC6

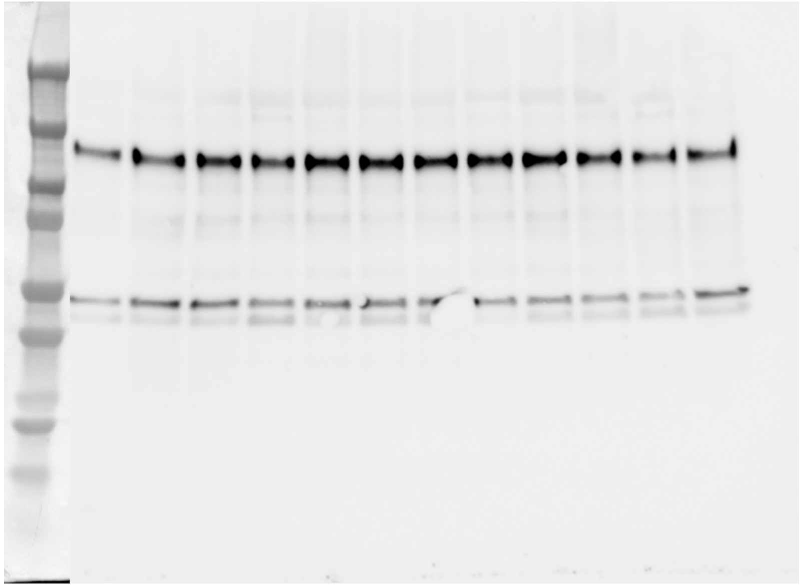

sFig. 4 – GAPDH

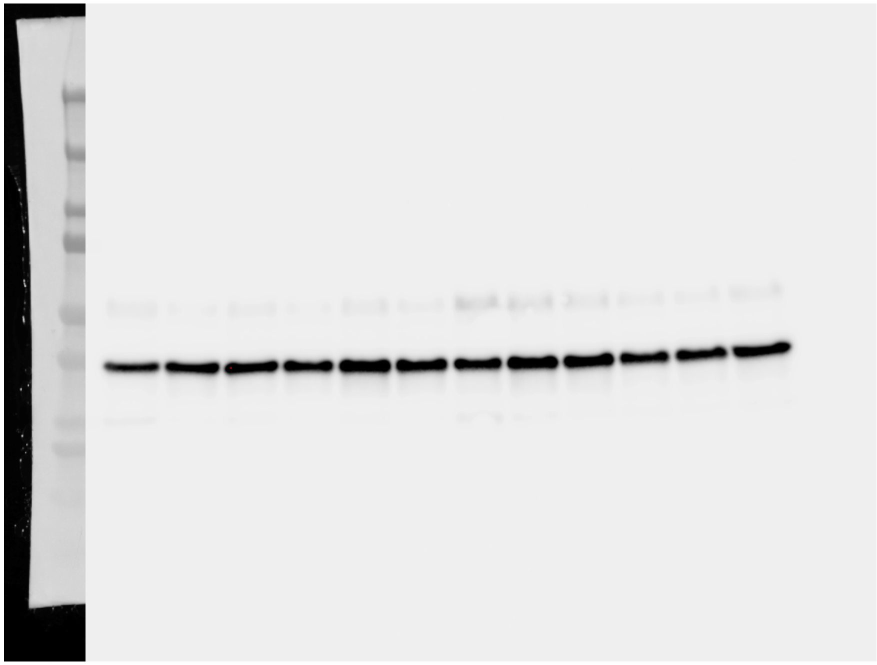

sFig. 4 – Total Tubulin

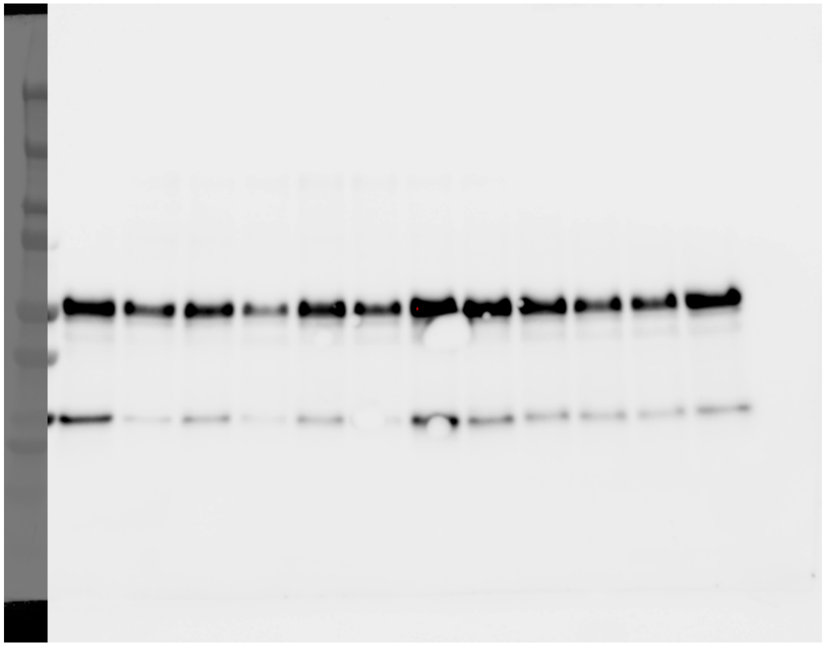

sFig. 4 – Acetyl-Tubulin

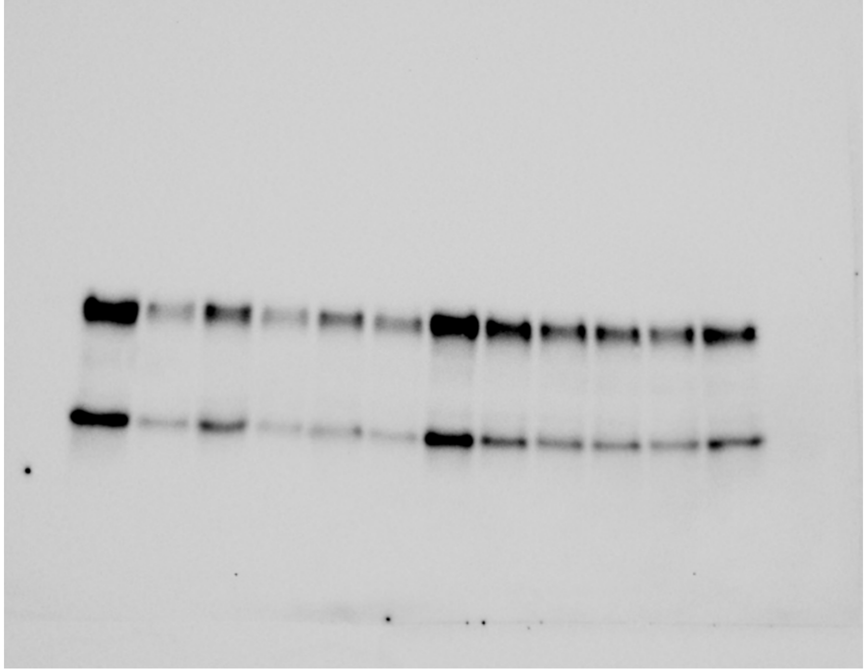

sFig. 5 – P-S11916

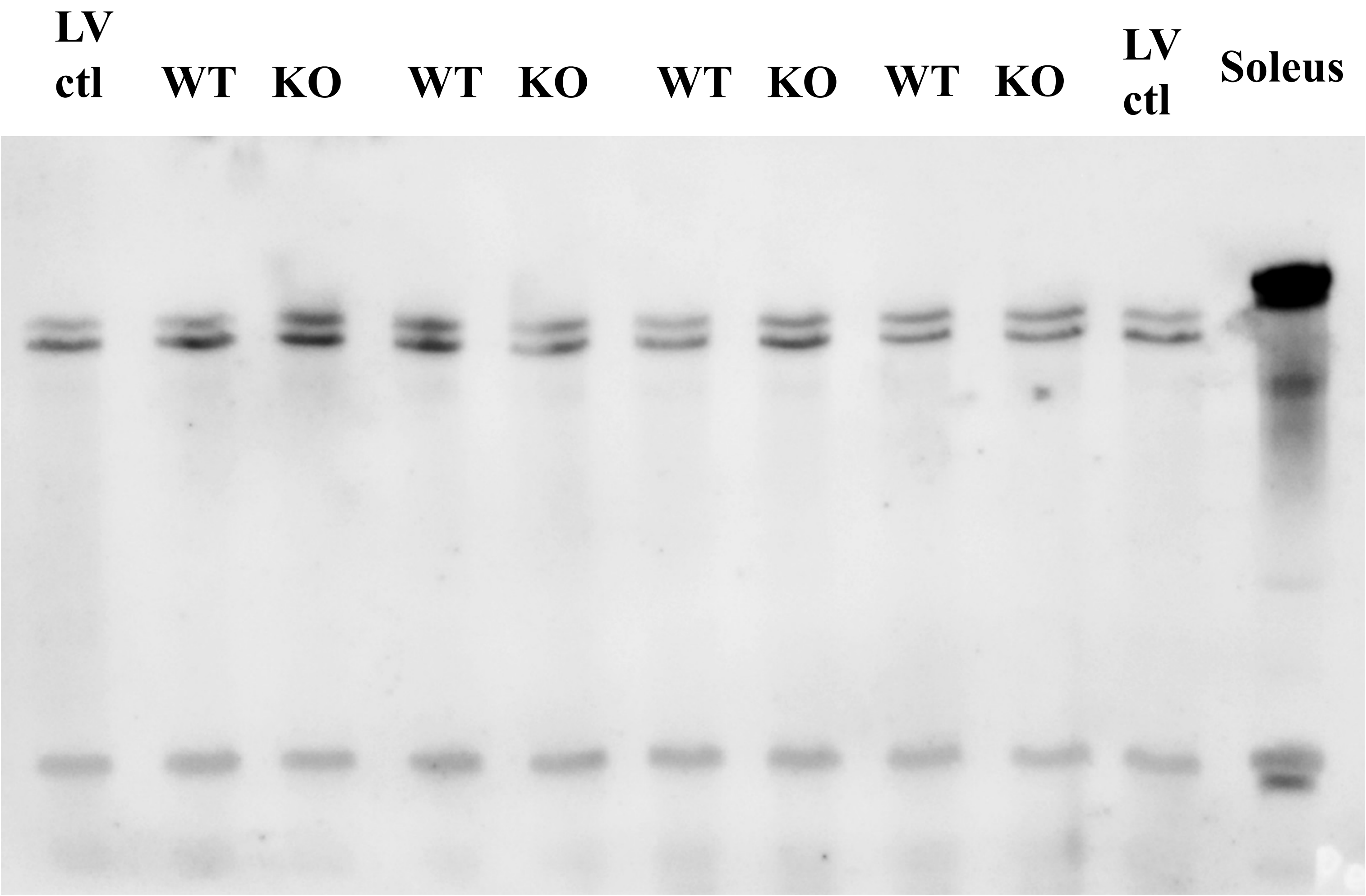

sFig. 5 – P-S12037

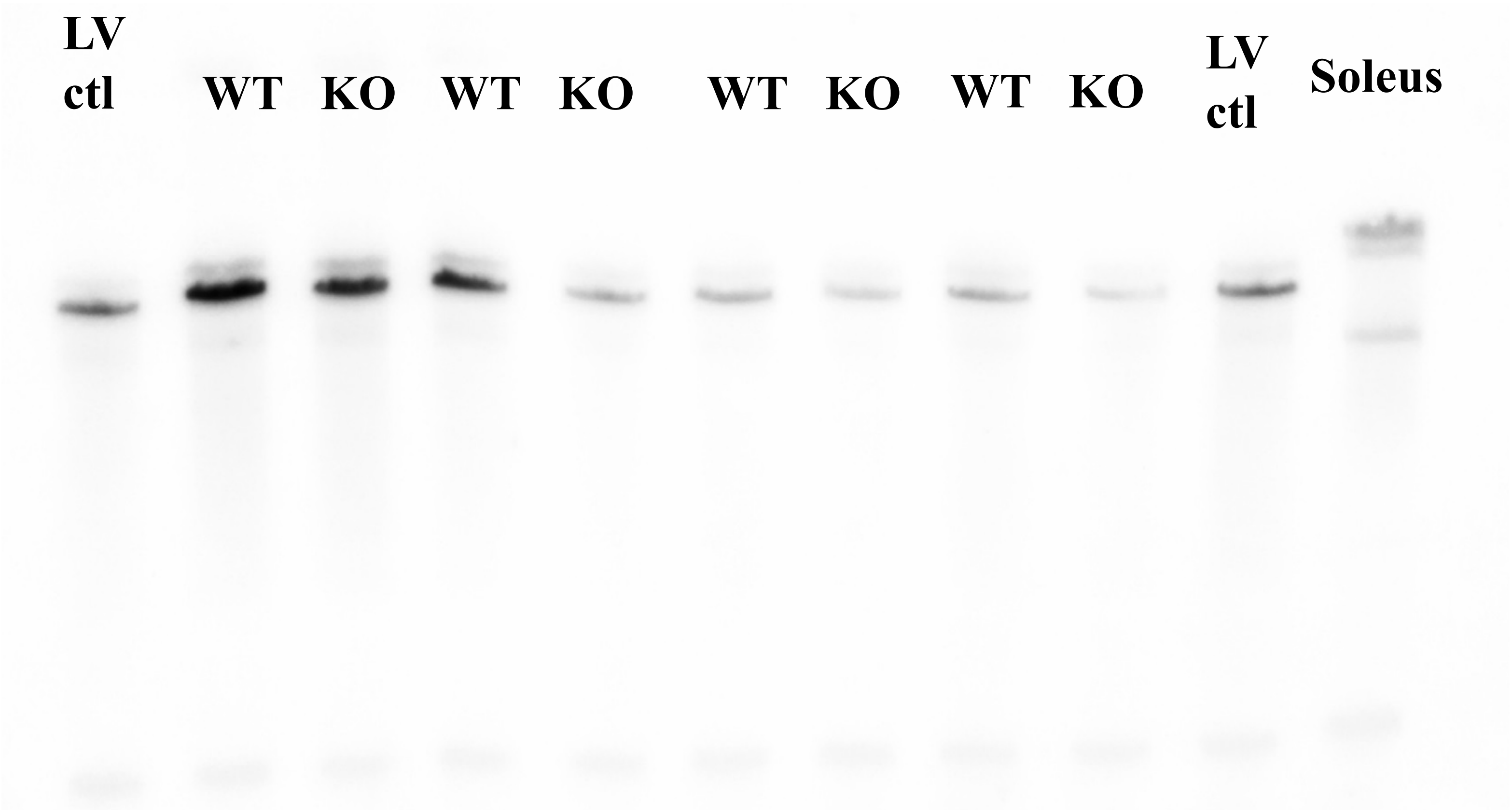

sFig. 5 – P-S3991

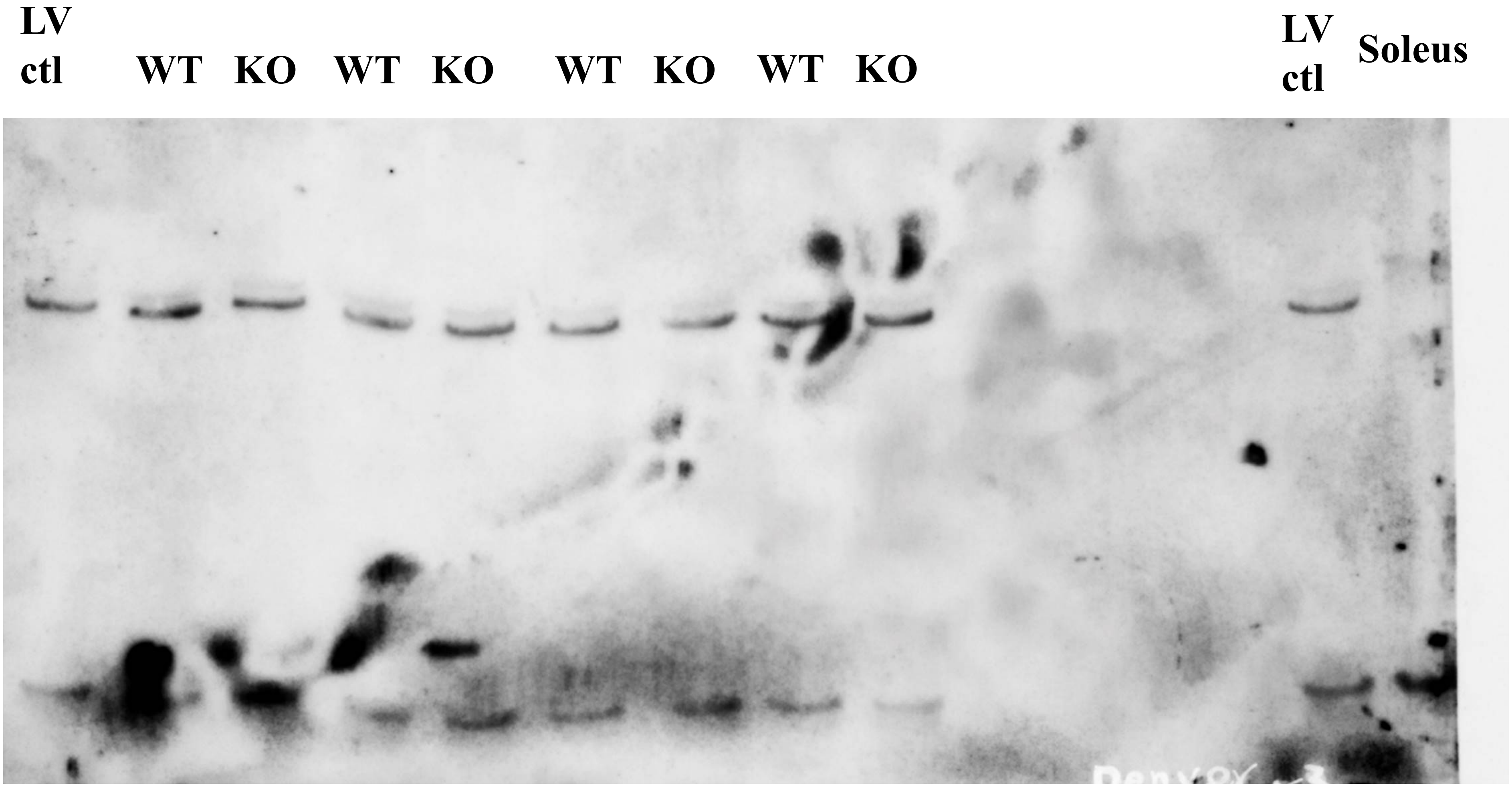

sFig. 5 – ZIZ2

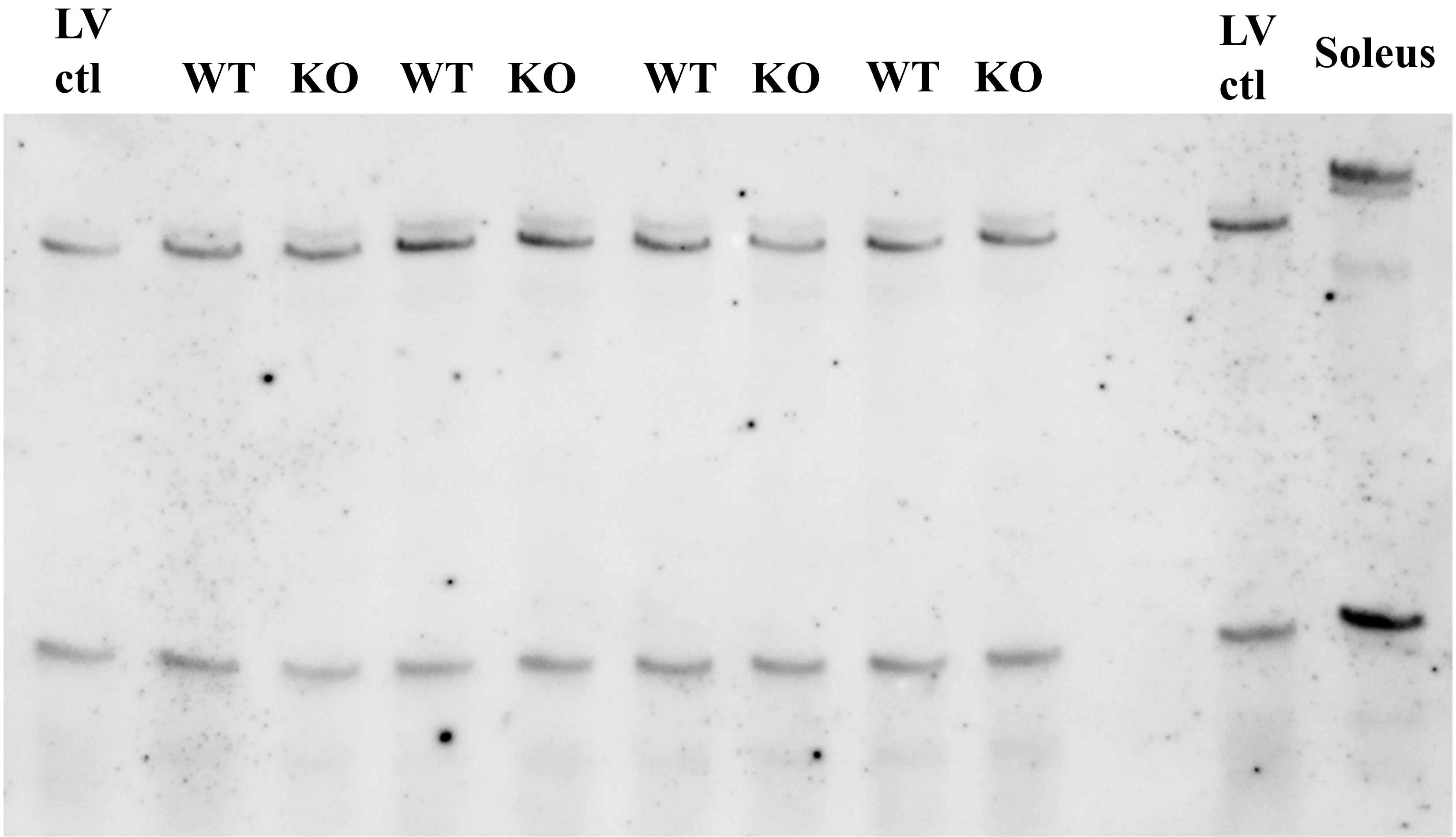

Titin gels are cut above myosin prior to blotting

sFig. 6 – Acetyl-Lysine Ab #1

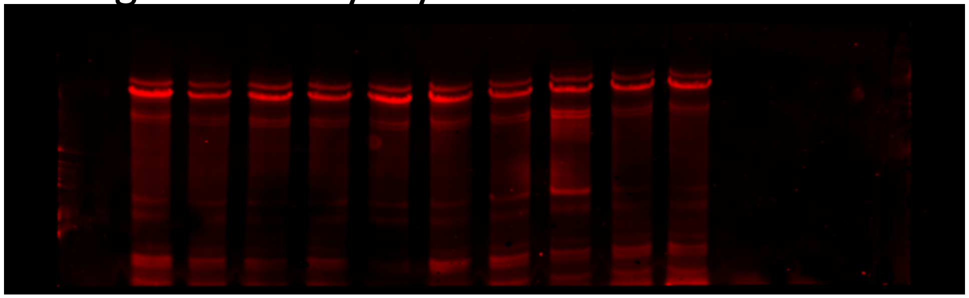

sFig. 6 – Acetyl-Lysine Ab #2

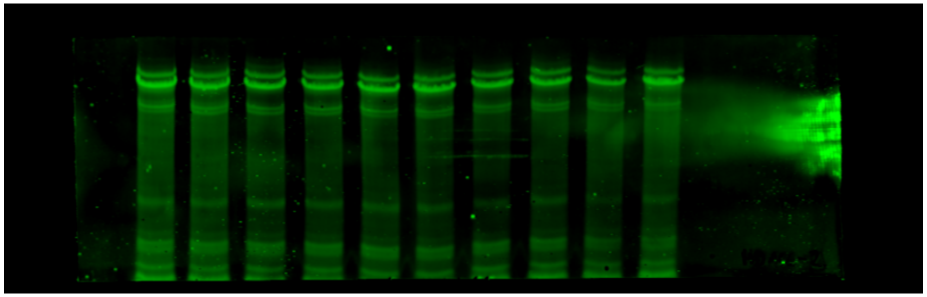

sFig. 6 – ZIZ2

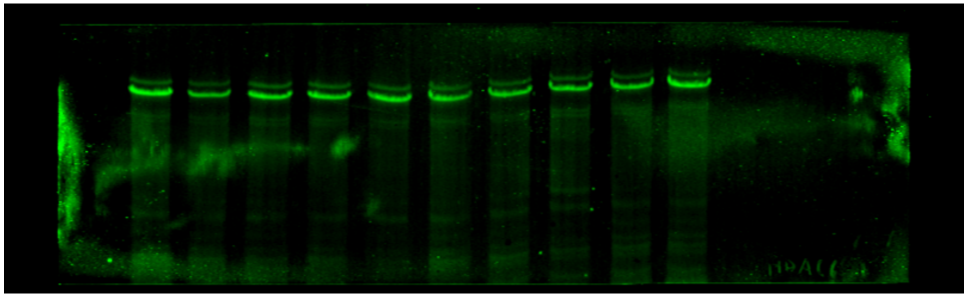

sFig. 6 – ZIZ2

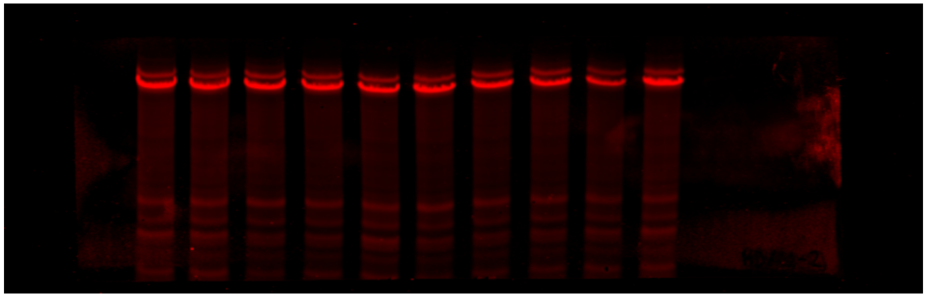

sFig. 6 – Merge

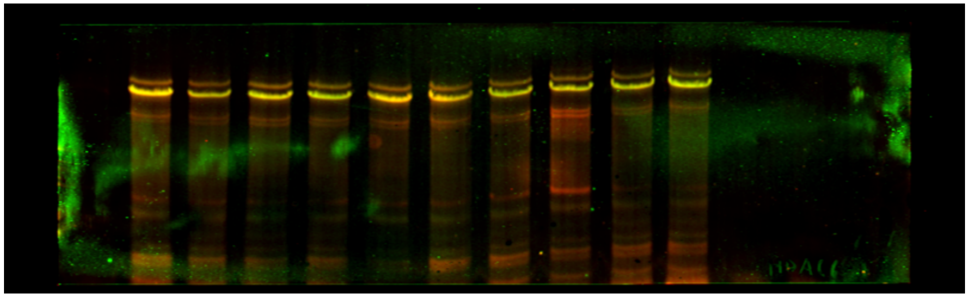

sFig. 6 – Merge

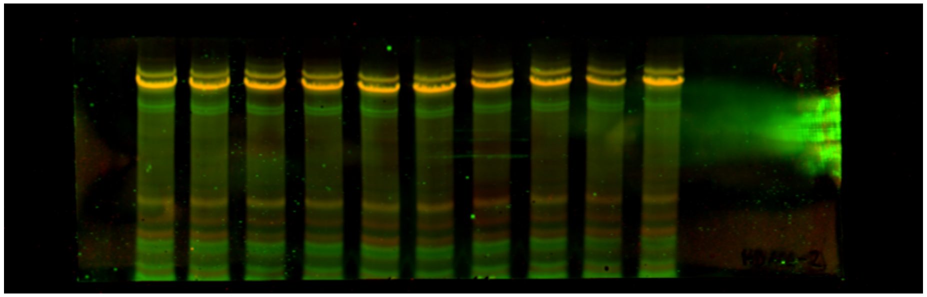

Supplement: Supplemental data [file jci-132-148333-s219.pdf]
